# Machine Learning in Preclinical Development of Antiviral Peptide Candidates: A Review of the Current Landscape

**DOI:** 10.3390/v18020260

**Published:** 2026-02-19

**Authors:** Hannah Hargrove, Bei Tong, Amr Hussein Elkabanny, Xiaohui Frank Zhang

**Affiliations:** 1 Department of Chemical and Biomolecular Engineering, University of Massachusetts Amherst, Amherst, MA 01003, USA; hhargrove@umass.edu; 2 Department of Biomedical Engineering, University of Massachusetts Amherst, Amherst, MA 01003, USA; btong@umass.edu; 3 Department of Chemistry, Massachusetts College of Liberal Arts, North Adams, MA 01247, USA; amr.ibrahim@mcla.edu

**Keywords:** antiviral peptides, artificial intelligence, machine learning, early-stage drug design

## Abstract

In the field of antiviral peptide (AVP) design, one of the most prominent limiting factors is the time and material cost required to perform the initial screening of novel AVPs. In particular, traditional target identification as well as traditional preclinical screening of novel drug candidates can be a very lengthy and expensive process. In recent decades, target identification and initial screening of AVPs has been increasingly carried out using machine learning (ML). The use of ML to initially screen potential interactions reduces the financial cost and lengthy time scale of preclinical AVP development, allowing for candidate peptides to be identified and screened faster, at a lower cost to both manufacturer and consumer. Additionally, the use of ML in generating and screening AVP candidates allows a more diverse chemical space to be explored than high-throughput screening methodologies allow. In silico generation and validation of AVP candidates also limits researcher contact with high BSL-rated viruses, thereby increasing the safety and accessibility of AVP design. This review seeks to provide a broad overview of the current uses of ML in early-stage AVP design, and to shed some light on the future direction of the field.

## 1. Introduction

Globally, communicable viruses remain a primary health concern [1,2], particularly in the wake of the global COVID-19 pandemic. Furthermore, there is growing concern about the global increase of drug-resistant viruses [3], which can give rise to drug-resistant viral infections. AVPs have potent activity, low cytotoxicity, broad-spectrum efficacy, novel mechanisms of action, and low likelihood of inducing resistance [2]. In addition to these attractive qualities, advances in peptide synthesis and production enable the development of custom peptides with increased stability, specificity and pharmacokinetics [2]. With all these points in mind, AVPs are a promising source of novel antiviral therapeutics.

The use of AVPs to treat viral infections began in 1951, when Groupé et al. observed the notable protective effect of viscosin on embryonated eggs, which were infected with infectious bronchitis virus, as well as a milder protective effect on influenza A suppression in mice [4]. Since that time, the interest in AVPs as potential therapeutic candidates has grown in popularity, with a marked uptick in activity starting in the early 2000’s [5]. AVPs are abundantly found in nature and have a critical role in the innate immune systems of many organisms. It is relatively simple to extract and purify AVPs from natural sources via various types of chromatography, such as HPLC [6], TLC-DB [7], and LC-MS [8], or they can be produced synthetically [2,9]. AVPs can demonstrate strong antiviral activity in a variety of contexts, which makes them highly viable as a treatment for persistent infections [3]. However, there are several known disadvantages to AVPs which must be accounted for. AVPs are prone to issues such as immunogenicity, proteolytic degradation before reaching the target, poor membrane permeability, and rapid in vivo clearance [2]. In light of this, an AVP-based therapeutic treatment needs an effective delivery strategy to be viable, such as encapsulation in liposomes or lipid nanoparticles, or conjugation with cell-penetrating peptides [2]. Advances in peptide design, delivery method, and understanding of viral biology will continue to attempt to address these obstacles adequately [5]. In parallel with the development of effective delivery pathways, the use of ML in initial peptide design and screening allows for potential AVPs to be optimized towards their intended target, as well as minimizing toxicity and predicted ADEs, so that the AVP underperforming is not the limiting factor in clinical AVP success.

As was briefly touched on, there are several AVPs and AVP-based therapeutics which have been approved for clinical use in a variety of antiviral applications [10]. In a drug development context, AVPs are a highly attractive candidate for novel therapeutics. They can originate from a wide variety of natural sources, which include plants, animals, and microorganisms, and can also be produced via peptide synthesis in a lab environment [2]. Furthermore, AVPs are favored by biopharmacologists for several reasons: AVPs exhibit high pharmacological activity, high specificity, low toxicity, minimal side effects, broad applicability, low rates of allergic reaction, low drug-drug interactions, and high diversity and modification ability [2,5,9]. Additionally, they typically have rapid onset of action and rapid exit from the system, which makes them very useful in treating acute viral cases [2]. Clinically, AVPs can be combined with additional therapeutic approaches to aid in overcoming antibiotic resistance, potentially leading to improved outcomes and reduced dosing requirements [2]. Furthermore, AVPs can also be used prophylactically to prevent viral infections in at-risk populations [2], typically in the form of topical application, mucosal delivery, or incorporation into prophylactic vaccines [2]. All of these critical applications make AVPs a very attractive candidate for a wide variety of pharmacological problems.

### 1.1. AVP Mechanisms of Action

Distinct virus entry mechanisms may require specialized modeling strategies if virus entry is dependent on characteristics external to the AVP itself. For example, the VEIP entry model is dependent on the sequence of the target protein as well as the peptide sequence to make accurate predictions—this is a specialized approach targeting virus entry inhibition. Other models, like iAVPs-Res-Bi, do not need data beyond what the peptide sequence (and sometimes structure) can provide. The need for external characteristics is very use-dependent—in general, the environmental variables should be as closely cataloged as possible, so that the model may make use of relevant variables to improve model performance. Ideally, a large feature set is available to then be reduced by recursive feature elimination, to determine the variables which are most critical to the predictive task. In general, ML modeling is a broadly applicable tool, but the individual models are almost always tailored to a specific task, and will underperform at other tasks. With this in mind, we will examine some well-known FDA-approved AVPs, which will help demonstrate some of the potential ways in which an AVP can inhibit viral infection.

The first FDA-approved AVP was Enfuvirtide [11], an AVP that blocked the entry of HIV-1 into host CD4 lymphocytes. Enfuvirtide works by associating strongly with the HIV glycoprotein GP41, which is critical to the viral fusion step. Since that single step is inhibited, the viral infection process cannot proceed, so the disease is mitigated. This is referred to as fusion inhibition, one of the many mechanisms by which AVPs can treat viral infections. While Enfuvirtide has a strong EC50 value against its intended target, it has experienced a lack of sweeping success because patients dislike injecting the medication, and some significant inflammation can occur at the injection site if used repeatedly [11]. Thus, other fusion inhibition drugs such as fostemsavir [12] are preferred, as they can be taken orally and have minimal side effects.

In recent years, several more AVP candidates have been proposed against a wide variety of viral targets [10,13,14,15,16,17,18,19,20,21,22,23,24,25]. Each of these candidates is capable of interfering with some phase of the virus life cycle in order to prevent successful viral infection. It is important to understand the desired mechanism of action when designing an AVP, so that a clear pathway may be targeted in early-stage AVP design.

External to the host cell, a peptide may cause spike inhibition (binding or coating the viral spike proteins so they are inhibited) [3]. This is seen in the action mechanism of Enfuvirtide, which works by associating strongly with the HIV glycoprotein GP41 [11]. As GP41 is critical to the viral fusion step, this effectively prevents further viral activity within the host. Likewise, the four-amino acid peptide GSRY has been observed to bind to the membrane protein of SARS-CoV-2, blocking membrane fusion in a similar way [5,26]. A peptide may similarly bind to a viral membrane receptor and cause co-aggregation of the virus [3]. For example, HD5 and RTD-1 bind to their viral targets (HIV and RSV, respectively), and cause the viral particles to clump together. Due to this clumping behavior, the virus is no longer able to penetrate the host cell, thus preventing further viral activity in the host [3].

In enveloped viruses, it is also possible for an antiviral peptide to disrupt the envelope of the virus, which renders it incapable of normal function [3]. For example, LL-37 binds and co-aggregates its target virus, causing it to form pores and rupture, likewise disrupting viral activity [3]. Some peptides have demonstrated an ability to destroy the capsid layer of non-enveloped viruses, such as cecropine B, CF17, and CAP37 [3]. This would similarly render the virus incapable of further activity.

At the cell surface, a peptide may compete directly with a virus binding pocket. This is the action mechanism of bLfcin, which prevents adenovirus binding to HEp-2 cells by direct competition with the HEp-2 binding pocket [3]. Alternatively, it may bind a neighboring receptor in some manner, which blocks the virus from binding the intended target [3]. Inside the cell, the AVP can affect other critical infection steps. LVLQTM is a six-acid peptide that acts as a pseudosubstrate, which blocks 2Apro and prevents viral replication of HRV and EV71 [3]. The LVLQTM peptide was modified so that it contained an electrophilic group, which allowed it to form a covalent bond with the active-site thiol and a benzyloxycarbonyl group. This enhanced its ability to enter the cell, so that it can access the protease to inhibit it [27]. Similarly, the peptide may inhibit translation or transcription, or it may release proinflammatory cytokines such as INFγ, which would slow or stop the cell replication cycle and inhibit the further spread of the virus [2,3]. Melittin works in this way by interfering with chemicals like ATPase, which are critical for fusion, thereby preventing HIV fusion [3].

### 1.2. Properties of Successful AVPs

The antibiotic effect of antimicrobial peptides (AMPs) is typically measured by assessing the efficacy of a given AMP at inhibiting bacterial growth [28]. However, since viruses can’t live outside of a host cell, viral replication inhibition can’t be observed in such a direct manner. Instead, the inhibition of specific virus-related processes or produced substances by AVP action must be measured, which allows for the indirect measurement of virus inhibition in the host. For example, measuring the amount of integrase, reverse transcriptase, protease, viral replication, cell fusion/entry rates, and plaque formation are all common tactics to assess the activity of an AVP [28]. By these measurement techniques, the highest virus-inactivating activity for AVPs has been reported against enveloped viruses such as SARS-CoV-2, HIV, HBV, HCV, HSV, and INFVA, with non-enveloped viruses demonstrating a higher degree of robustness towards most AVPs [3,10]. This is owed to the noted success of envelope-rupturing AVPs against enveloped viruses, which naturally would be ineffective against non-enveloped viruses. Instead, non-enveloped viruses must be inhibited by other methods, such as co-aggregation or virus-specific receptor inhibition, rather than direct virus inactivation.

Although AVPs exhibit diverse viral inhibition mechanisms, there are several shared characteristics that define an AVP. In general, AVPs are widely recognized as peptides 8–15 AAs in length, which are predominantly positively charged due to a high presence of lysine and arginine in the AVP primary structure [3,29]. Successful AVPs also have a higher observed frequency of alanine, glutamine, and tryptophan than non-AVPs, and a lower frequency of proline and threonine [28,30]. The positively-charged nature of most AVPs, largely due to a strong presence of lysine and arginine, allows them to more easily interact with negatively charged surfaces, such as anionic cell membranes, phospholipid viral envelopes, and bacteria [3,29]. Most highly effective AVPs are also enriched with large acidic amino acids at their N-terminal regions and a mixture of large polar/non-polar acids at their C-terminal regions [31]. It has been observed that AVPs with cationic and amphipathic characteristics, which have positive net charges are essential for antimicrobicity [29], and it has also been observed that hydrophobicity seems to be a key component for AVPs targeting enveloped viruses [29]. Furthermore, the strong presence of lysine in AVPs is hypothesized to be due to lysine’s ability to enhance electrostatic interactions between the AVP and negatively charged surfaces, such as anionic cell walls and phospholipid membranes [29]. In the case of large DNA viruses, the presence of serine and threonine is critical to the phosphorylation of serine/threonine kinases [29].

The length of the AVP chain also significantly affects its activity and stability. It has been observed that shorter peptide lengths may penetrate membranes more effectively [32], while longer peptides may demonstrate increased specificity and stability [33]. Therefore, optimizing the length of an AVP chain to a specific use case is vital for the optimization of therapeutic outcomes.

In addition to the presence of key amino acids and optimization of peptide length, there are certain physicochemical properties which may have a significant effect on AVP activity. For example, successful AVPs demonstrate high hydrophobicity and amphiphilicity when compared to non-AVPs [28,34]. Furthermore, factors such as the electronic charge of the AVP, the environmental pH and temperature, and the number of metal ions present in the primary sequence may affect the activity and stability of an AVP [3,35].

At the secondary structure level, there is some evidence that the formation of more α-helices may lead to an increase in AVP activity [36], with many successful AVPs taking on an α-helix secondary structure. This structure may allow the AVPs to better interact with their associated target proteins. AVPs also demonstrate a lower frequency of polyproline helix II coil formation [28], which may suggest a limited or nonexistent presence of proline [37]. Therefore, integration of structure characteristics as well as sequence-derived variables is likely to be critical to effective AVP classification.

Overall, although there is a high degree of diversity within the population of known AVPs, most will exhibit a positive charge, with a strong presence of arginine, lysine and hydrophobic amino acids, with a probable α-helix secondary structure.

## 2. Foundations of Machine Learning Methods

When discussing ML, it is important to distinguish between unsupervised versus supervised techniques. While both have useful applications, they are each better-suited to distinct tasks. Unsupervised learning is ML that does not require labeled data, instead using a large body of unlabeled data to form observations about the overall qualities of the dataset. This can include applications such as genome-wide association studies (GWAS) to identify critical virus receptors in a host population, or assess the shared characteristics of a population of interest. Alternatively, supervised learning requires labeled data, and is used to form predictions based on input data that is provided. This includes applications such as predicting the binding affinity or likelihood of a clinical adverse drug effect (ADE) based on some patient criteria, as well as physics-based predictive tools like AlphaFold3 [38]. To improve the comprehensive understanding of the models reviewed in this paper, the authors will present here a brief overview of the core methods of unsupervised and supervised ML.

### 2.1. Unsupervised ML

Within the field of unsupervised ML, there are several prominent applications in preclinical drug development. Primarily, unsupervised ML is used for exploratory purposes. One such technique of exploration is dimensionality reduction. This is the use of unsupervised ML to visualize high-dimensional data, such as unlabeled population data from a known risk population of a certain disease. This can be used to identify promising candidate leads or target receptors [39]. In a similar use case, clustering is when unsupervised ML is used to separate a large group of molecules into similar sub-groups based on input variables such as chemical properties. This is used to identify novel structures which may be effective against a virus based on structural similarity to molecules which are known to have antiviral activity.

When using dimensionality reduction, the primary goal is to find a low-dimensional vector representation of high-dimensional data [39]. This can be done by Bayesian or frequentist approaches, and within frequentist approaches there are linear and nonlinear methods [39].

Within a Bayesian approach, the Bayesian theorem of probability would be used to form observations about an unlabeled dataset. This includes techniques such as probabilistic principle component analysis, Gaussian process latent variable models, and variational autoencoders.

A linear frequentist approach is used to develop a linear projection of high-dimensional data into a low-dimensional space. One of the most common methods of linear frequentist dimensionality reduction is principal component analysis [39], where a large body of characteristic data is used to form observations about the behaviors of particles within the sample set. Likewise, a non-linear method would seek for a non-linear projection of high-dimensional data into a low-dimensional space. This includes such techniques as tSNE, autoencoders, and UMAP [39].

Clustering is used to identify relevant groupings among a certain population of interest. This is done by observing patterns within the data, and splitting the larger population into groupings such that the instances within a certain group are more like those within the group than instances outside the group [39]. This can be incredibly useful in preclinical tasks such as identifying patient risk populations or forming observations about the qualities of successful antiviral drug candidates. Some of the most common methods of clustering are k-means clustering, hierarchical clustering, and Gaussian mixture models [39]. Each of these methods uses a slightly different unsupervised ML configuration to group instances within a dataset.

### 2.2. Supervised ML

Supervised learning is distinguished from unsupervised learning by its ability to make predictions based on a set of input data, as well as by its requirement that the input data be labeled in some way. Supervised learning can be broken down into two phases: inference (training) and decision-making (testing) [39]. In the inference phase, the model conducts some form of self-optimization to produce the best predictions over the training set, and during the decision-making phase the optimized model is applied to a distinct testing set. The model’s ability to correctly predict the labels on the testing set is then used to evaluate the model’s performance. Additionally, the target labels can be either discrete (a classification task) or continuous (a regression task). Both classification and regression tasks can be accomplished via a variety of ML methods, each of which has its own qualities and drawbacks.

Historically, cutting-edge supervised ML methods have included RandomForest (RF) and Support Vector Machine (SVM) models as the method of choice. More recently, neural networks have overtaken the presence of RF and SVM in many supervised tasks. Shallow neural networks (NNs), which have a single hidden layer, are a good tool for solving problems of mid-level variable complexity, but they may struggle to discern complex behavior within the larger set [39]. A commonly-used type of NN is a multi-layer perceptron (MLP), which tends to perform well on large datasets. For more complex applications, deep neural networks (DNNs) may be preferred. These are neural networks with multiple hidden layers, which are capable of forming more complex inferences about the interaction of various input variables and their effect on the predicted outcome. DNNs have several advantages over other ML models: they can easily model several behaviors at once (multitasking), and may generate novel chemical features based on encoded input data [39]. They also facilitate the inversion of quantitative structure-activity relationship (QSAR) models (i.e., designing molecules straight from the model in a generative capacity). However, in spite of the manifold advantages of DNN, there are some drawbacks to the model. For example, the complexity of the DNN configuration may demand more tuning to improve model performance. Furthermore, the computational cost can be large, and the results may be difficult to interpret. Additionally, the best use of DNNs is when there is a large body of validated data to draw from during the training and testing phase – without this large dataset it is unlikely the model will be able to capture many complex behaviors in their entirety [39].

Similar to DNNs, convolutional neural networks (CNNs) were originally designed for vision tasks, such as image recognition [39]. In a drug design capacity, candidate molecules can be represented as a graph of data, and interpreted in a manner similar to how an image may be processed. Also in this category are graph neural networks (GNNs), which function very similarly, but may be able to take a more general grid of points as input data than the CNN model. For example, a set of atoms and the connections between them may be acceptable for each data point when training a GNN [39].

Slightly more complex than DNNs, there are also models that use recurrent neural networks (RNNs). These were originally used for natural language processing, as is seen in web browser auto-complete search functions. These RNN models have feedback connections that make the network aware of time-dependent factors. A good example of this type of use case is the Elman network [39]. In this capacity, back-propagating through long sequences or time-dependent inputs can lead to the propagation of minute imperfections in the data. This is referred to as vanishing or exploding gradients [39]. To mitigate this issue, gating mechanisms become necessary. These safety blocks are built into the code to prevent the excessive effect of older time-dependent variables on the overall model performance. The introduction of gating leads to several distinct models, such as single-directional gated recurrent unit (GRU) networks, bidirectional gated recurrent unit (BiGRU) networks, and long short-term memory (LSTM) networks [39]. Recurrent neural networks also rely on the ability of the input data to be interpreted as an input string, similar to how language processing works. For chemical design tasks, the most common string notation for molecules is the SMILES sequence notation, although other sequence representations are present in the literature [39].

If the sequential processing method of RNN is swapped out for running operations in parallel, we arrive at the implementation of transformers. The transformer model is built from scaled-dot-product attention layers that produce activations for every element in the sequence. Each layer of a transformer usually contains several parallel layers. These are referred to as multi-head attention layers, and they are what allow the transformer model to multitask. The data is encoded via the first layer of the transformer, and then alternating attention layers and feed-forward layers allow the data to move through the network of weights, finally exiting the model through a decoding layer, which outputs prediction probabilities. Within the scope of the basic transformer, more advanced transformer architectures have also been established. This included the Bidirectional Encoder Representations for Transformers (BERT), as well as several generative pre-trained transformers (GPTs) [39].

Aside from GPTs, there are several other model classes which can generate novel data, including autoencoders, variational autoencoders, GrammarVAE, Constrained Graph VAE, and Generative Adversarial Networks [39].

Having briefly outlined the fundamentals of the wide variety of ML models that may be discussed in this review, it is the hope of the authors that the exact mechanism of the various models that are discussed may be better understood.

## 3. Aspects of Early-Stage Screening

**Figure 1:**
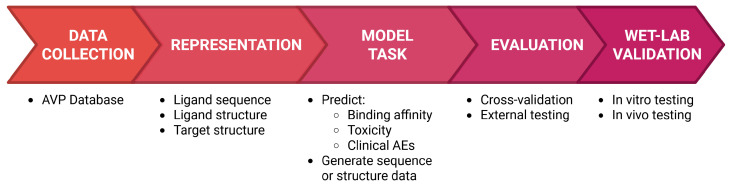
AVP ML workflow roadmap. Overview of an AVP machine-learning pipeline from data and feature representation to modeling tasks (activity, toxicity, ADE, generation), evaluation (cross-validation/external tests), and wet-lab validation (in vitro/in vivo). Image produced in Biorender.

In the early stages of AVP development, several areas of research can be accelerated and diversified with the assistance of ML. A basic AVP ML development workflow is shown in Figure 1, which illustrates progression from data collection and feature representation through performing and validating relevant model tasks, finally resulting in some wet-lab validation through in vitro and in vivo methods. This review focuses on the variety of model tasks that can be performed by ML to accelerate the early development of AVPs. These tasks include initial target identification, candidate sequence generation, on/off target binding affinity prediction, and prediction of in vivo toxicity as well as clinical ADEs. The traditional modalities of each of these research areas will be discussed, and then the current methods for implementing ML in these areas will be juxtaposed against the traditional methods for qualities such as time and material costs.

### 3.1. Lead Identification

The first step of drug discovery, and thus AVP discovery as well, is lead identification. As most drugs preserve at least 80% of the scaffold of the initially identified lead [40], this is a critical first step in the AVP development process. In the case of AVPs, this is the discovery of a peptide which has the potential to be highly successful against a preselected target.

Historically, leads have been identified via genetic, cellular or animal models to identify and validate a target compound, followed by iterative large-scale screening to improve the candidate’s bioactive properties before in vitro testing [40,41]. In a modern context, this initial testing is often accomplished by high-throughput screening (HTS).

**Figure 2:**
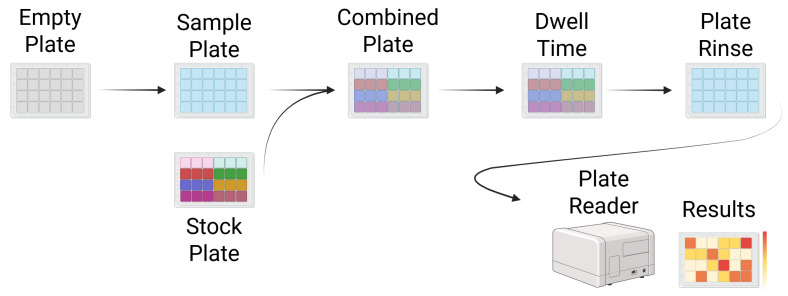
Schematic representation of a high-throughput screening experiment process. An empty plate is seeded with a sample, then combined into a sample + stock solution. The plate is incubated, rinsed, and read for fluorescence. Image produced in Biorender.

In the HTS process, a robotic system is used to conduct immunoassay testing at a rapid rate, producing a large quantity of data about sample compounds in a short period of time [42]. For example, an automated system could be used to conduct an ELISA binding assessment between a lead compound and dozens of common drug targets, which is illustrated in Figure 2.

As shown in Figure 2, a typical HTS experiment involves the use of liquid handling and processing robots to precisely pipette a sample of interest into a well-plate, whereupon a highly concentrated stock plate is used to test the sample plate for suspected binding via immunoassay testing. After testing yields quantifiable results, interesting binding events (“hits”) can be re-examined, refining the information gained at high speed [42]. Although the schematic representation in Figure 2 is a 24-well plate, typical HTS plates are usually in multiples of 96, with many HTS machines capable of testing hundreds of binding combinations at once [42]. This highly-efficient mechanization of a standard immunoassay allows lead compounds to be explored at a much higher rate than human-conducted immunoassays, and was revolutionary when it first emerged [42].

The HTS experimental model was made possible by the emergence of automated handling systems in the 1950s and 60s [42], and was a critical research breakthrough when it was introduced. Around the same time, most researchers switched to cell-based assays and molecular biology techniques, which also greatly improved accuracy and relevance of data [42]. The benefit of HTS handling systems over a human operator is that the use of automated control equipment allowed sample handling and analysis to be performed without manual interference. This reduces the opportunity for error, thus increasing the quality of the experimental results [42].

This HTS assessment of what molecules were likely to bind to a given target was and is critical to early-stage antiviral drug development, as most drugs preserve 80% or more of the initially-identified lead scaffold [43]. A main component of the HTS process naturally involves producing the compounds before testing; this usually takes several weeks to make and deliver compounds for HTS, along with being reasonably expensive.

In drug screening, HTS allows rapid testing of large compound libraries to identify potential drug candidates [42]. Furthermore, the integration of fluorescence techniques, as well as the integration of highly sensitive assays like ELISA, allows for the results of an HTS experiment to be easily interpretable and highly precise, provided the experimental conditions are carefully selected [42]. In combination with the use of HTS, large-scale peptide libraries can be cloned or synthesized and combined with cell surface display technologies to rapidly obtain highly specific peptides for new drug development, sometimes in a matter of weeks [40,42], thus further increasing the scope and utility of HTS.

Traditional screening methods before HTS, such as manual screening and biochemical assays, required extensive time and labor costs, which made the concept of doing large-scale screening unfeasible [42]. Conventional pre-HTS screening methods utilized one of three techniques: administering compounds directly to animals and observing the pharmacological effects, extracting human patient serum for drug testing, or simple biochemical analysis such as chromatography [42]. Each of these approaches has valuable applications: Animal model screening offers greater physiological relevance than simple cell-based assays, and human sample screening better predicts human effects over animal models. However, both human and animal models are not possible via HTS, therefore HTS is focused on biochemical assays [42]. In a typical drug discovery pipeline, the order of testing may be that preliminary large-scale screening is conducted via HTS, with only the promising candidates proceeding to animal and then human testing.

Certain limitations still persist in HTS-based lead identification. Primarily, the requirement of a broad and diverse sample set being physically present is a limiting factor [42]. The fact that the samples must be synthesized before testing results in a high initial cost of materials. Furthermore, the results of an HTS study are limited to the chemical space defined by the samples under consideration [40,42], which can be limiting in terms of chemical diversity of resultant candidate peptide structures. Additionally, use of HTS as a lead identification step involves a high rate of false positives, and relies on the necessity of simplistic physical models due to the nature of HTS testing capabilities [42]. For example, screening for a highly effective AVP can be conducted via HTS by measuring binding affinity to a critical receptor, but this simplistic measurement of binding may not accurately reflect the efficacy of the lead candidate in an in vivo setting. All these factors in combination point to HTS being a highly valuable first-step identification tool which has some practical limitations.

When discussing the material cost of peptide development, it is important to contextualize how much it costs to produce peptide-based therapeutics as opposed to alternatives, such as small molecules and monoclonal antibodies. A report from Pharmaceutical Outsourcing [44] indicates that the average total development cost to get a peptide-based drug to IND submission is $1–2 million. In comparison, generic development plans from SRI Biosciences estimate the cost of small molecule development to be $3.5–6.0 million [45], and the cost of monoclonal antibody development to be $19.2–26.3 million [46]. It is generally agreed that monoclonal antibodies are much more expensive to produce than small molecules or peptides [44,46,47], due to their larger complex structures. However, the difference in cost between small molecules and peptides can be dependent on the specific candidate—in addition to the earlier figures mentioned, it has also been posited that peptides are generally more expensive to produce than small molecules [44,48]. This cost differential is largely attributed to specialized methods of peptide production, such as solid-phase peptide synthesis [44]. This would position small molecules as favorable over peptides in terms of cost: however, production of the active pharmaceutical ingredient (small molecules or peptides) is estimated as 1–3% of the total IND cost [44,48], which would make any production cost difference between small molecules and peptides negligible in the scope of the total pre-IND costs. Further, by integrating ML in the early candidate selection and screening processes, this cost can be minimized to only include wet-lab testing of predicted successful candidates, thus reducing the total pre-IND costs further by ensuring the number of failed wet-lab experiments is minimized.

While HTS has made large-scale screening accessible to the modern researcher, the integration of ML can improve the speed and accuracy of lead identification even further [42]. Computational approaches combined with vast chemical on-demand libraries can greatly expand the chemical space, if the accuracy of the approach is high enough [40]. A computational approach may also allow lead performance to be predicted before synthesis—this makes it possible to determine which lead candidates are worth producing and analyzing further, thus saving time and material cost in not having to synthesize every lead candidate before initial screening [40]. In addition to the simple acceleration of identification and validation of candidate AVPs, it may also be possible for in silico methods to more adequately explore a diverse chemical space, as ML models may observe patterns of AVP behavior which may be difficult to assess via traditional means. Due to this fact, computational approaches promise to improve over HTS in terms of cost, speed, need to produce sufficient quantities of protein, the effort of miniaturizing assay formats while maintaining experiment integrity, and reducing false positive and false negative rates [40].

The earliest computational approaches include ligand-based QSAR, structure-based docking, and early machine learning techniques, such as SVMs [40]. More recently neural networks, particularly DNNs, have been integrated into the lead identification step. As deep learning can form more complex interactions between input variables, it is markedly more successful than preceding ML techniques in a variety of lead identification tasks [40]. In fact, modern machine learning approaches have been able to achieve a degree of computational accuracy, which allows them to potentially replace HTS as the initial step of lead candidate identification [40], provided that the right conditions are met.

When considering whether ML should be used in the lead identification step, a couple of points must be examined. First and most critical, computational approaches to lead identification are limited by the need for a large quantity of highly dependable training data [40]. In light of this, it is important to consider whether such data is accessible to the researcher before ML-based lead identification should be attempted. The amount of data which is considered to be ‘sufficient’ for an ML training and testing configuration varies widely depending on the type of ML being used, as well as the exact application being considered. For example, if a receptor being screened for binding peptide candidates is highly polymorphic, then a larger quantity of data will be needed to ensure that all relevant binding behaviors are being accounted for in the model. Alternatively, if the receptor in question has a very low degree of polymorphism, an ML model may possibly be trained to a high degree of accuracy on a relatively small amount of data.

In addition to the issue of database size, it must also be taken into consideration that despite excellent benchmark accuracies, computational approaches still have a modest discovery accuracy [40]. In light of this, it is important to assess the accuracy, precision and recall of the ML model being used to try to assess the amount of false positives and false negatives which are likely to be assigned. Further, it is critical to test lead candidates further via traditional lead identification assays once they have been identified by ML techniques. In spite of this consideration, it may still be valuable to utilize ML in the first stages of lead identification, as it will minimize the amount of false leads which need to be synthesized and tested in vitro.

### 3.2. In Vitro Toxicity Screening

The resultant effect of off-target binding events can encompass a wide range, from a benign, low-level side reaction up to an ADE, which triggers abnormal behavior in the cell. The capability of a drug to cause harm is referred to as drug toxicity, and is most frequently a result of off-target binding [49]. In an AVP development context, off-target binding is when the candidate peptide binds to a protein other than its intended target. As of 2018, approximately 21.7% of compounds entering the clinical phase of drug development fail due to drug toxicity [50], so it is evident how critical the early establishment of potential toxicity is to effective drug development. Machine learning models which assess drug toxicity fall into one of two categories: models which directly predict toxicity from test subjects (toxicity prediction), and models that predict off-target binding in order to minimize toxic side reactions (off-target binding prediction). Historically, assessment of off-target binding and toxicity is done either in vitro by probing the critical organ cells [51], or in vivo by probing animal models [52].

Current investigational new drug application for initiation of clinical phase I study requires extensive toxicity studies in at least three different animal species [49]. However, there is increasing evidence that non-toxicity in vitro and in vivo is not consistently predictive of ADEs at the clinical dose [49]. Further, the results from toxicity testing on animals are highly inconsistent predictors of toxic responses in humans, which has led to the FDA Modernization Act in the United States [49]. Therefore, while cell-based and animal-based study is important to remove strongly toxic compounds from consideration, it is more situationally relevant to attempt to minimize clinical ADEs, rather than toxicity alone.

### 3.3. Clinical Adverse Event Screening

Part of the ongoing development in attempting to improve the early stages of AVP development is developing effective methods of predicting and reducing ADEs in end-use patients. As was briefly mentioned, this is typically done by one of two methods: investigating the binding of drugs to unintended targets (off-target binding affinity prediction), or investigating the resultant toxicity effects of the drugs in a test subject (toxicity prediction). Since the predictive models can draw on publicly available existing data, they can be used to screen candidates for high toxicity early in development, with only the most promising peptides going to production for further testing.

As was also mentioned, however, the prediction of in vitro or in vivo toxicity is not necessarily a confirmation or denial of a clinical ADE occurring. Therefore, it is critical to develop methods for predicting clinical ADE behavior for a given drug structure, rather than trying to predict toxicity alone. Some groups are doing this by using large volumes of patient data on clinical ADE occurrences in clinical testing. This allows for previous ADEs, along with the structural data and dosage of the trial drug, to predict whether an ADE is likely based on the drug structure. If this can be accomplished, then a novel drug could be screened in advance of phase I testing to minimize future ADE occurrences. This would be of great use, both in reducing harm to the patient population by predicting harmful ADEs before they can occur, and in reducing the time and material cost spent in pre-clinical development.

## 4. AVP Features to Consider for Model Construction

To form a robust predictive model, the appropriate features must be examined and supplied to the model. In an AVP development context, this includes but is not limited to features such as molecular descriptors, previous test results on the AVP candidate, molecular fingerprints, and a wide variety of sequence-derived chemical features [53]. Molecular descriptors include such parameters as molecular weight, number of carbon atoms, number of single, double, and triple bonds, number of hydrogen bonds, and so on. Similarly, test results on the AVP candidate include values such as the oil-water partition coefficient, Moore refractive index, dipole moment, hydrophobicity, and so on. Additionally, test results can include any prior in vitro or in vivo testing that was done on the training data peptides, such as the results of a binding affinity assay. Molecular fingerprints, as the name would suggest, are a representation of the training molecules’ unique molecular structure, which can be expressed through a variety of techniques such as SMILES fingerprinting or, in the case of AVPs, a single amino acid abbreviation of the peptide sequence.

There are also a wide variety of sequence-derived variables, other than the direct representation of the sequence itself, which can be used to train an ML model. A list of the most common sequence-derived features is available in Table 1.

In addition to all these variables, it is also possible to incorporate some aspects of the target protein to improve the predictive capabilities of the model. In an AVP context, this may include incorporating a fragment of the viral envelope protein sequence, or a fragment of the protein structure of the targeted virus-critical receptor. By integrating elements of the target protein into the prediction of AVP candidate behavior, the prediction can sometimes be improved to increase the model’s ability to generalize across several polymorphic targets [28]. This may be especially useful in AVP applications, as if an existing AVP exists which is effective against one strain of a virus, and then a new strain emerges, it may be possible to predict whether the AVP will also be effective against this new strain by adjusting the composition of the viral fragment which is being fed to the model [28].

As can be easily seen, there are a wide range of potential input variables which can be used to train an AVP candidate assessment ML model. Indeed, ML developments of late tend to focus on increasing the number of available variables to test with [54]. However, endlessly increasing the number of input variables can be detrimental to model performance—by utilizing multiple inputs which are interdependent on each other, model performance tends to suffer [54]. Instead of using all possible input features, a procedure known as feature reduction should be conducted to optimize the model. This is a process where a series of ML experiments are conducted to determine which features are the most critical to model performance, typically by measuring the Mean Decrease of Gini Index (MDGI) when the variable is included or removed. By using feature reduction, a researcher can ensure that their model does not include useless or redundant variables, thus offering the best chance of the ML model’s success [54].

## 5. Current Leading ML Models

To produce a successful AVP, a target protein critical to the virus infection cycle must be identified, so that an AVP can be designed to bind strongly to that target with a high degree of selectivity, while minimizing toxicity and ADE risk. Therefore, the design of each new AVP ML model is highly dependent on the target protein and the location of that target protein within the cell. In a machine learning context, the more tailored a task is, the more likely that the model is to be highly accurate. However, if a task is too specific, there may be too little data to train a successful model. To make a useful ML model, it is necessary to split the difference between the two: the task must be as specific as possible, while also being general enough that a sufficient body of data can be secured for training and testing.

Having discussed at length the way in which ML may be used to accelerate and optimize the preclinical stages of AVP development, the current cutting-edge published prediction and classification models will now be discussed, with attention paid to the merits and disadvantages of each model. There are several published models which do not have a publicly-available internal architecture—these are so-called “black box” models, as they function as a whole unit that can’t be assessed internally. For this review, the authors will not be discussing the performance of black-box models, as it is not productive to discuss their efficacy without in turn being able to examine why they are effective. Instead, the authors will focus on models that have clear, publicly available internal architecture and a high degree of explainability, so that the models’ successes may be used to the best advantage for future researchers. For additional clarity, models will also be split into sections by functionality: either binding affinity prediction, toxicity prediction, clinical ADE prediction, or the generation of novel AVP candidate sequences.

### 5.1. Binding Activity Prediction

Of the AVP preclinical testing and validation tasks, the most common by far is the prediction of the binding activity of a candidate peptide. This may be because HTS allows for the rapid generation of a wide variety of high-quality training data to be acquired rapidly, facilitating the formation of robust training and testing sets. As such, there are several published models with a high degree of success in their prediction tasks. Each will be discussed in terms of their model architecture and the performance of the model, as well as the steps each of the requisite authors took to optimize the model structure towards the goal task.

#### 5.1.1. iAVPs-ResBi

**Figure 3:**
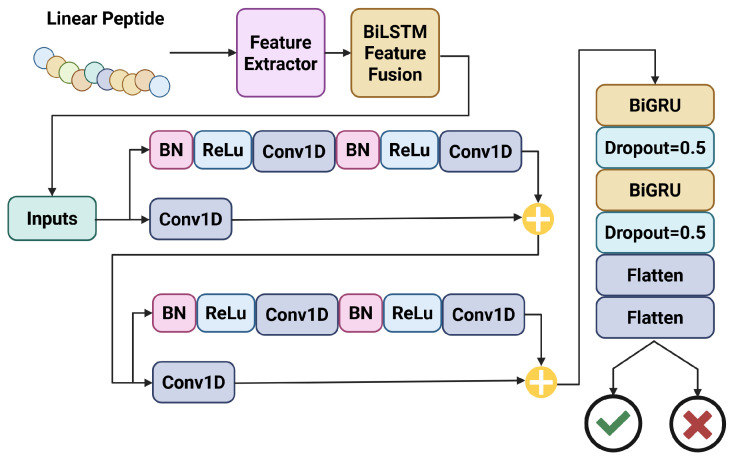
A schematic representation of the model architecture for iAVPS-ResBi as presented by Ma et al. The linear peptide data is fed through a feature extraction module, and the resultant features are then fused into a suitable vector for ML by BiLSTM processing. The data then cycles through numerous Batch Normalization (BN), Rectified Linear Unit (ReLu) activation functions, and 1-dimensional convolutions (Conv1D), before being processed by two rows of BiGRU modules. The results from the BiGRU modules are then flattened, and the outputs are used to determine whether the peptide is predicted to bind the target. Figure produced in Biorender.

Ma et al. proposed a binary AVP prediction model which is capable of determining whether an input peptide sequence will have strong antiviral capabilities, based on a variety of sequence-derived input factors [9]. Such input factors include k-spaced amino acid pairs (KSAAP), encoding based on group weight (EBGW), composition of the N- and C-termini (N5C5), and composition, transition, and distribution (CTD) based on physicochemical properties. The model fuses these features by bidirectional long short-term memory (BiLSTM), whereby any redundant features are removed from consideration, and discrete variables are merged into a robust input vector for classification. The authors also demonstrate that fusing the input features dramatically improves model performance. Using this fused feature vector, the model then implements a residual neural network (RNN) with bidirectional gated recurrent units (BiGRU) to perform classification. The final model can achieve a strong predictive performance, with accuracy, sensitivity and specificity all being 0.95. The precise data flow and model architecture for iAVPS-ResBi is shown in Figure 3.

Ma et al. place a specific emphasis on the model’s ability to predict whether peptides will have antiviral activity against zoonotic viruses, including Zaire Ebola virus (EBOV). It is also noted that as the number of layers in the depth of the iAVPs-ResBi network increases, the identification accuracy reaches a saturation point, and then performance rapidly degrades beyond that point. This is in line with what is known about other deep learning model architectures.

What is particularly interesting about the structure of iAVPs-ResBi is the utilization of an RNN in combination with BiGRU. An RNN utilizes a hop-connected network structure, where the input is both fed through the neural network layer for prediction and also carried alongside the layer, to be considered alongside the output of the layer in question. This allows the model to consider the layer output alongside the layer input. This helps to minimize variable explosion, where multiple compounding differences lead the model to the incorrect conclusion. In combination with BiGRU units, which allow the model to perform forwards and backwards learning steps, this makes the iAVPs-ResBi model very stable, which, in combination with the high level of accuracy, makes this a reliable initial model for antiviral peptide screening.

#### 5.1.2. Virus Entry Inhibition Peptide (VEIP) Prediction Model

**Figure 4:**
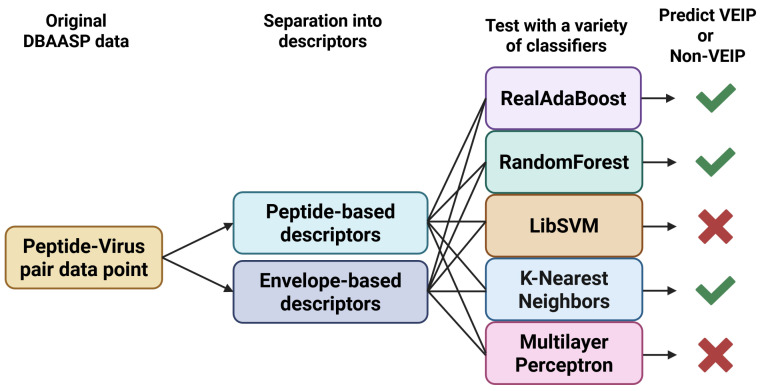
A schematic representation of the model architecture for the VEIP prediction model proposed by Vishnepolsky et al. From linked peptide-target datapoints, peptide-based descriptors and viral envelope-based descriptors are extracted. Then, a variety of ML classifiers are assessed for their ability to generate accurate predictions based on this input data. RealADABoost was found to have the best performance, with a prediction accuracy above 0.90. Image produced in Biorender.

There is a particular subset of AVP which is known as virus entry inhibitory peptides (VEIPs). These are AVPs that are focused on preventing the initial virus fusion and infiltration step. One such common method of prevention is a peptide disrupting the membrane of an enveloped virus, which results in the virus rupturing and becoming incapable of further progression. Most effective VEIPs form alpha-helices, which seem to have a structure which is particularly suited to envelope disruption [28]. Vishnepolsky et al. [28] were the first to predict VEIP envelope disruption ability based on features derived from both the VEIP candidate protein sequence and the viral envelope’s protein sequence. A schematic representation of the VEIP model’s internal architecture is depicted in Figure 4. Samples were selected from DBAASP for inclusion in the training and testing of the model by noting either a binding affinity less than 50 nM to the target protein of choice (positive sample) or a binding affinity greater than 100 nM to the target of choice. This yielded an unbalanced set, so further negative sample points were collected by sampling non-AVP peptides from UniProt. This resulted in a total dataset of 246 positive datapoints and 246 negative datapoints, of which Vishnepolsky et al. allocated 73% for training and the remainder for testing. VEIP sequence-derived features included normalized hydrophobic moment (NHM), normalized hydrophobicity (NH), net charge (NC), isoelectric point (IP), linear moment (LM), cyclic linear moment (CLM), propensity to in vitro aggregation (PA), propensity to ppII coil (PII), angle subtended by the hydrophobic residues (AH), amphiphilicity index (AI), propensity to disordering (PD), amino acid composition (AAC), and peptide length (PL) [28]. In addition to these VEIP sequence-derived features, some features were also extracted from the protein sequences of the target viral envelopes, with the original authors highlighting the integration of viral envelope structure characteristics into the predictive models.

Five models were tested for VEIP envelope disruption prediction, including RealAdaBoost (RA), LibSVM (SVM), and generic forms of RF, KNN, and MLP models. Ultimately, it was found that the RA model was the most effective of the five, with a VEIP classification accuracy of 0.89 over the test set. Sensitivity and specificity were not directly reported for comparison. Although this is a reasonable success rate, Vishnepolsky et al. emphasize the small amount of the publicly available VEIP training data, most of which came from DBAASP [55], and recommend that the model be re-trained with more VEIP data, should the opportunity arise in the future.

One of the most critical components of the VEIP prediction model is the inclusion of viral envelope protein characteristics in the prediction model. This allows for future viral mutations to be accounted for by altering the input for viral characteristics, and could allow the model to predict strong VEIPs against novel mutations. This is cited by the original authors as being highly useful in the event of a breakout of a novel virus strain [28], and is a critical building block for the advancement of AVP prediction models that can be adapted to novel problems.

#### 5.1.3. FIRM-AVP

Feature-Informed Reduced Machine Learning for Antiviral Peptide Prediction (FIRM-AVP) is a model proposed by Chowdhury et al. [54], which utilizes the most critical sequence-derived features of an AVP candidate to predict whether the candidate will be a successful AVP or not. Specifically, the initial design of the model involved filtering all possible sequence-derived features to only include features which were independent and non-redundant in the model’s architecture.

This concept of feature reduction is based on the machine learning principle that having codependent input variables fed into a model may harm the performance of the model, therefore reducing the input variables to only include variables which are independent is valuable towards increasing model performance. For FIRM-AVP, the features of importance were determined by calculating the Mean Decrease in Gini Index (MDGI) and Pearson’s Correlation Coefficient (PCC) for each feature. Following this, the authors used a technique known as recursive feature elimination (RFE) in combination with an SVM structure to determine the order of importance of each feature. The final number of ordered independent features was 169, down from 649 features.

**Figure 5:**
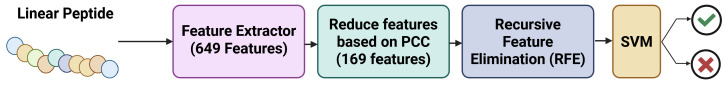
A schematic representation of the model architecture for the FIRM-AVP prediction model proposed by Chowdhury et al. From linear peptide sequence data, 649 possible sequence-based features were extracted. Feature reduction based on best Pearsons Correlation Coefficient (PCC) brought the number of features down to 169, and then recursive feature elimination (RFE) was used to weight the features by importance to model performance. The resultant optimized feature data is then fed to an SVM model, which makes the final prediction as to whether the peptide can bind a target. Image produced in Biorender.

The authors also tested multiple versions of FIRM-AVP, which included an RF, an SVM, and a DNN model, ultimately revealing that the SVM performed the best, with a model accuracy/sensitivity/specificity of 0.92/0.93/0.91. The FIRM-AVP authors were also able to demonstrate that FIRM-AVP has a higher accuracy than similar models with no feature selection, further demonstrating the importance of down-selecting to include only independent non-redundant features. A schematic representation of the data processing steps and model architecture for FIRM-AVP is shown in Figure 5.

The FIRM-AVP authors also note specifically that the DNN model performed markedly worse than the SVM and RF models, and hypothesized that the difference in performance ability could be due to the extremely small dataset available. This is owed to the fact that while DNN model configurations can form complex interaction relationships between input features, they also require a large volume of data to fully encompass the space of possible outcomes. Since the training set is so small, it is most likely not possible to adequately train a DNN to fully encompass the space of possible AVPs, as there are most likely distinct AVPs in the testing set that are highly dissimilar to those present in the training set. This would reduce the accuracy of the DNN model, as is seen here. This demonstrates that while DNN is a powerful tool when a large volume of data is available, it is not necessarily universally applicable.

The authors of FIRM-AVP also note that the AVPpred benchmark dataset, which has been used as a benchmark dataset for AVP prediction training for the last decade, has not been altered since the original publication of AVPpred in 2012 [56]. The FIRM-AVP authors call for broadening and deepening this initial dataset, so that a larger volume of AVP binding data may be available to the scientific community.

#### 5.1.4. Comparison of Models

Both iAVPs-ResBi and FIRM-AVP use AVPpred, which is extremely dated at this point as it was last updated in 2012. Conversely, VEIP uses a subset of DBAASP, which was up to date when VEIP was produced in 2023. The VEIP dataset has some overlap with AVPpred, but does not include all the AVPpred virus targets. There are also virus targets in the VEIP dataset which are not well characterized by AVPpred. With this in mind, it is necessary to consider the biases associated with each dataset before discussing the merits and limitations of the models in comparison with each other.

**Figure 6:**
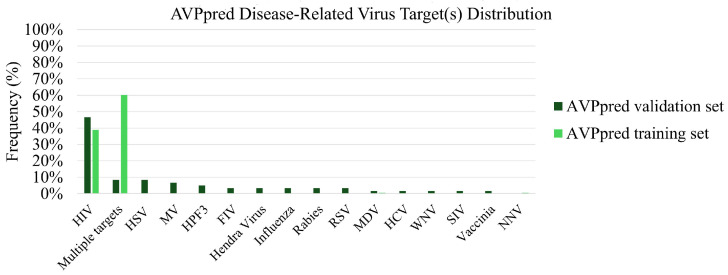
A bar chart showing the distribution of datapoints over possible disease-related targets in thetraining and testing subsets of the AVPpred dataset.

**Figure 7:**
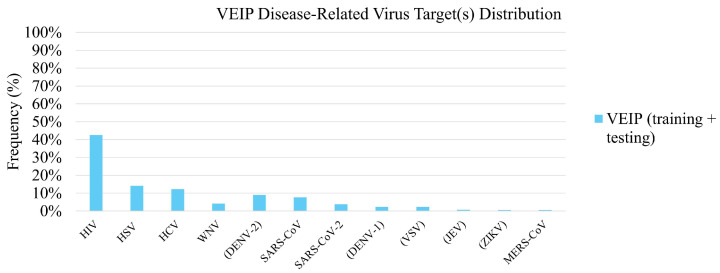
A bar chart showing the distribution of datapoints over possible disease-related targets in thecombined training and testing dataset for VEIP.

The AVPpred and VEIP datasets both have a strong bias towards HIV- and HSV-targeting peptides, as can be seen in Figure 6 and Figure 7. AVPpred also has a preference towards generic AVPs which are successful against multiple targets. In the AVPpred subset of peptides which are successful against multiple targets, there is a relatively even distribution of peptides targeting HIV, HCV, WNV, DENV, measles virus, RSV, and paramyxoviruses in some combination. Notably, the VEIP dataset contains peptides targeting the Zika virus and MERS-CoV, which AVPpred does not, and AVPpred has several disease targets that are absent from the VEIP dataset, including RSV and influenza. With this in mind, picking a model for predicting binding affinity of a known AVP candidate should be informed by the disease target, as well as model performance—if the disease target is Zika virus, or the envelope of an enveloped virus, the VEIP model is preferred, since it is highly specialized to that task and is likely to generate good predictions. Likewise, if the target is INFVA, the iAVPs-ResBi and/or FIRM-AVP models may be used—both perform well, and if both can be used to predict binding affinity, the results may be combined to ensure the researcher makes a well-informed decision about whether a peptide candidate should progress to further screening or not. Additionally, the researcher should bear in mind that both AVPpred and VEIP are heavily biased towards HIV and HSV. Therefore, they are most likely to be successful in predicting HIV- and HSV-targeting peptides as having high binding affinity. This may result in a larger number of false positives for HIV- and HSV-targeting peptide candidates, and perhaps a higher number of false negatives for non-HIV/HSV-targeting peptide candidates. This shortcoming would be improved by increasing the depth and breadth of publicly-available, experimentally-validated AVP binding affinity data.

In addition to potential biases based on disease target, there is some suspicion of potential bias based on candidate peptide length. The exact lengths of the VEIP peptide datapoints are only reported as being between 9 and 40 amino acids long, but the exact length of each entry of AVPpred is listed, so the possibility of length-based bias can be explored.

**Figure 8:**
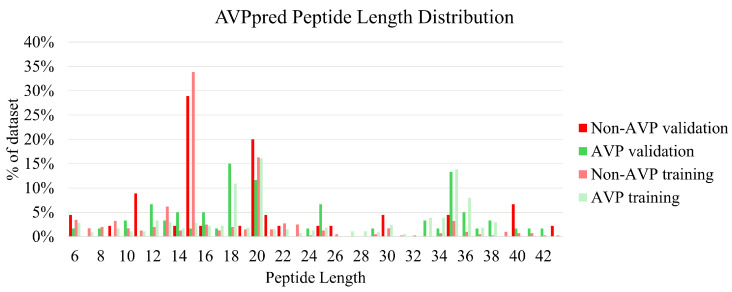
A bar chart showing the distribution of peptide lengths over the training and testing subsets of the AVPpred dataset.

As shown in Figure 8, there is a noticeable difference between the most common lengths of non-AVP and AVP peptides: namely, most of the non-AVP peptides are 15 or 20 amino acids long, while many AVP peptides are 18, 20, or 35 amino acids long. This may cause some slight bias in terms of peptide length, where models trained on the AVPpred dataset may be prone to predicting that shorter peptides are not AVPs, and/or predicting that longer peptides are AVPs. However, this does not seem to be overly effecting performance, so the effect on overall model predictive capability for iAVPs-ResBi and FIRM-AVP is likely negligible.

When using ML models, data labeling noise should be considered. In an ML context, label noise refers to incorrect labeling of datapoints (labeling an AVP as non-AVP or vice-versa). This would naturally reduce the performance of models trained on that data. However, this is not anticipated to be a highly prevalent issue in this context—the binding affinity databases used by all three cited models are based exclusively on experimentally-validated IC50 and EC50 data. Therefore, the likelihood of improper labeling and label noise is negligible in this instance.

When comparing iAVPS-ResBi and FIRM-AVP, iAVPs-ResBi uses exclusively sequence-derived features, while FIRM-AVP incorporates secondary structure features as well as sequence-derived features. In spite of the inclusion of structural characteristics, FIRM-AVP performs slightly worse than iAVPs-ResBi, though it still performs quite well. This difference could be due to the use of BiGRU layers in iAVPs-ResBi, which the original authors indicate is meant to reduce overfitting and thus improve overall model performance. This supposition is supported by the fact that an implementation of iAVPs-ResBi without the BiGRU layer performs about as well as FIRM-AVP. Therefore, it is likely that the BiGRU is assisting in preventing overfitting for iAVPs-ResBi, which would improve overall performance over FIRM-AVP. VEIP performs well over its test set, with a reported accuracy of 0.89. However, the VEIP model does perform notably better over the training set—this could indicate that the model is slightly overfitted to the training data, which would reduce its performance on the test set.

Overall, when selecting a binding affinity model for candidate AVP screening, the most critical consideration is the target virus. If the target is contained in AVPpred then the iAVPs-ResBi model should be attempted first, and then validated with the FIRM-AVP model. If a potential candidate has favorable predicted outcomes on both models, then there is reasonable evidence to justify progressing the candidate to further screening. If the target virus is not within the AVPpred dataset, but is within the VEIP dataset, then the VEIP model is a very good candidate for predicting peptide binding affinity. In the event that the target protein is not contained within AVPpred or the VEIP dataset, then use of these models may still be attempted, but the results should be interpreted with a high degree of skepticism: depending on the target and how closely it resembles similar targets in the training set, the models may or may not be able to correctly predict the binding affinity.

### 5.2. Drug Toxicity Prediction

When developing a successful AVP candidate, one of the most crucial elements of design is ensuring that the toxicity of the therapeutic dose is minimized. To do this, the toxicity of the AVP must be assessed at the in vitro and in vivo level before it can progress to a clinical study. However, these tests are time-consuming and expensive, and they are also poorly suited to high-throughput screening as they involve live cells or animal models. Therefore, the potential for in silico toxicity screening is naturally a hot topic in the field of peptide-based drug development—if highly toxic compounds can be discarded from consideration in silico, then only peptides with a low probability of being toxic need to be progressed to in vitro or in vivo toxicity screening, thus reducing the labor and expense of the project and accelerating the identification and discarding of toxic peptide candidates.

Over the last couple of decades, several attempts have been made to assess the toxicity of peptides at the in silico level. As the field of peptide design is much broader than the field of antiviral peptide design, there is a wide array of models which are designed to identify toxic peptides. For this review, we will discuss the peptide toxicity prediction models published within the last five years, along with the benefits and potential limitations of each.

#### 5.2.1. ATSE

**Figure 9:**
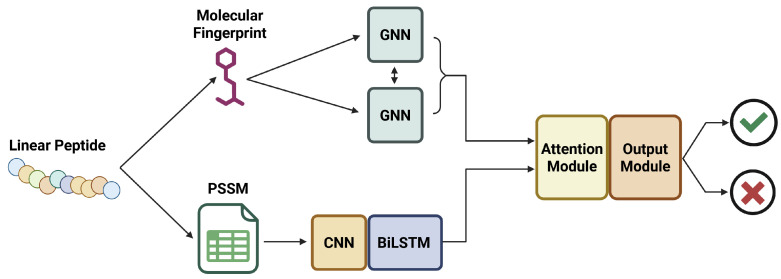
A schematic representation of the model architecture for ATSE. The sequence processing module processes input peptide sequences into molecular graphs and position-specific scoring matrices (PSSMs) for each sequence. The molecular graphs and PSSM matrices are then fed forward to GNN modules and a CNN-BiLSTM module, respectively. Finally, the outputs from the GNNs and the CNN-BiLSTM are fed into an attention module, which determines feature performance for peptide toxicity. Finally, the attention module feeds forward into the output module, which makes the final determination as to whether the peptide is toxic or not. Figure produced in Biorender.

While there are some prior models to predict toxicity, ATSE [57] is the first to use an ensemble graphical neural network (GNN) in combination with Bidirectional Long-Short-Term Memory (Bi-LSTM) structures to enhance the toxicity prediction capabilities of the model. The ensemble has four distinct segments: (i) a sequence processing module for constructing molecular graphs and evolutionary profiles from peptide sequences, (ii) a feature extraction module to analyze the graphical representations of the peptides and determine the most relevant graphical features, (iii) an attention module to optimize the weighting of the features on ensemble output, and (iv) an output module that determines whether the peptide is toxic or non-toxic, using the optimized features as determined by the attention module. A graphical representation of this ensemble workflow is shown in Figure 9.

When it was first published, ATSE was a revolutionary model configuration because it was capable of interpolating and optimizing graphical features with only the peptide sequences as a starting point. By comparison, prior models relied on user-defined optimized feature selection. The ability of ATSE to self-optimize was a revolutionary stepping stone in the field of peptide toxicity prediction, and resulted in a model that has a high performance capability. The published version of the model reported a sensitivity of 0.965, a specificity of 0.940, an accuracy of 0.952, and a Matthews Correlation Coefficient of 0.903. In the application of peptide toxicity prediction, it is generally considered to be the most important to maximize sensitivity, as this ensures that the number of false negatives (toxic peptides falsely predicted to be nontoxic) is minimized. For complete explanations of sensitivity, specificity, and accuracy, please refer to the Machine Learning Performance Metrics section. Maximizing sensitivity is a crucial aspect of toxicity prediction, as the overall goal is to mitigate harm caused by toxic peptide candidate drugs. Maximizing the sensitivity will ensure that the number of false negatives is kept to a minimum, but should also be weighed against other metrics like ACC and MCC, to ensure that the model is not becoming too cautious.

#### 5.2.2. ToxIBTL

**Figure 10:**
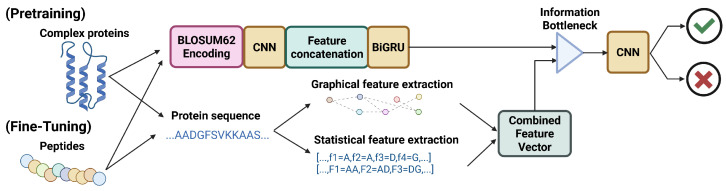
A schematic representation of the model architecture for ToxIBTL. In the pretraining step, large proteins are dually fed into a BLOSUM62 encoder and a protein sequence encoder, which then are used to determine a wide array of sequence-based features and learning-based pattern recognition. These features are then minimized at an information bottleneck and fed into a CNN, which makes the final determination as to whether the protein is toxic or nontoxic. This pretraining step allows the model to form appropriate weights for predicting protein toxicity, and then in the fine-tuning step, shorter peptides are used to tailor the model to be able to predict the toxicity of peptides. Figure produced in Biorender.

Following the success of ATSE, many authors of the ATSE research article were involved in a further exploration of in silico peptide toxicity prediction, ultimately inventing the model ToxIBTL [58]. Much like ATSE, ToxIBTL utilizes graphical representations of peptide sequences coupled with evolutionary information to represent peptides, and also utilizes both GNNs and CNN-Bidirectional Gated Recurrent Unit (BiGru) architectures, which allows the model to form local and long-term patterns, much like the implementation of GNNs and CNN-BiLSTM in ATSE. The most intriguing modification to ToxIBTL that sets it apart from its predecessor ATSE is the implementation of transfer learning—by allowing the model to learn patterns of toxicity from longer protein sequences, and then fine-tuning the model on short peptides, the model can gather a broader understanding of the qualities that make a peptide toxic or non-toxic by applying the patterns it learned from the protein sequences. A schematic representation of ToxIBTL is shown in Figure 10.

As shown in Figure 10, much of the model architecture from ATSE has been retained—the initial modules of feature extraction, graphical representation and GNN analysis, and CNN-BiGRU/BiLSTM remain largely intact. However, it must be observed that rather than directly conducting all training on the peptide dataset, the larger protein toxicity dataset is first used to train the model to recognize toxic and nontoxic amino acid sequence patterns. Then, the smaller peptide dataset is brought in to fine-tune the model on peptide toxicity, further improving model performance. The finalized ToxIBTL model was able to achieve a sensitivity of 0.963, which is comparable to ATSE, and a specificity and accuracy of 0.954 and 0.960 respectively, which is a statistically significant improvement over ATSE. Recall that sensitivity may be used as an indication of the model’s ability to minimize false negatives, and specificity is an indication of the model’s ability to minimize false positives. What these performance outcomes demonstrate, therefore, is that ToxIBTL is equally capable of minimizing false negatives as ATSE, and is slightly better at minimizing false positives (predicting nontoxic peptides as toxic). This is indicative of the model’s increased predictive capability, which is largely owed to the transfer learning step which is applied to ToxIBTL, which was not present during the training of ATSE.

#### 5.2.3. tAMPer

**Figure 11:**
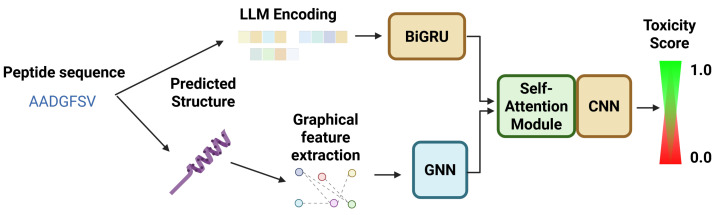
A schematic representation of the model architecture for tAMPer, sourced from the original manuscript by Ebrahimikondori et al. First, the peptide structure is predicted from the amino acid sequence. Then, the sequence and the graphical representation of the predicted structure are fed to the Bi-GRU and GNN units, respectively. Once the predictions from these two models have been made, the outputs are combined and fed into a self-attention model, which finally predicts both the peptide’s toxicity and its secondary peptide structure characteristics.

Following the successful release of ATSE and ToxIBTL by Wei et al., the next logical step was the integration of three-dimensional structure data along with the encoded peptide amino acid sequence. Ebrahimikondori et al. proposed a novel model, tAMPer, which integrates underlying amino acid sequence information alongside a graphical representation of the three-dimensional peptide structure as predicted by ColabFold. In that graph, nodes and edges represent amino acids and spatial interactions, respectively. The authors tested tAMPer over the same dataset as was used in ToxIBTL, and they also integrated their own data on AMP hemolysis. A representation of this model architecture is shown in Figure 11.

There are notable similarities between the structure of tAMPer and that of ATSE and ToxIBTL, namely the use of Bi-GRU and GNN units. However, tAMPer has an additional data preprocessing step, the generation of a graphical representation of the peptide structure. This yields a modest improvement in F1-score over ToxIBTL (about 0.03), and although the authors do not report the exact metrics of accuracy, specificity, and sensitivity for comparison, it must be observed that the integration of structure data yields at least a slight improvement in model performance. This is particularly interesting, as these results indicate that in silico values may be integrated into an ML model without the model’s performance being harmed by the inclusion of “artificial” data, provided that the necessary steps are taken to ensure that there is no leakage of data between the ML model in question and the source of the in silico generated data. In this case, the ColabFold model and the tAMPer model rely on distinct datasets, so there is no concern of data leakage here, and the integration of in silico ColabFold data improves the overall performance of tAMPer, over both the standardized benchmark data and the in-house AMP hemolysis data that the tAMPer authors reference.

#### 5.2.4. ToxinPred 3.0

**Figure 12:**
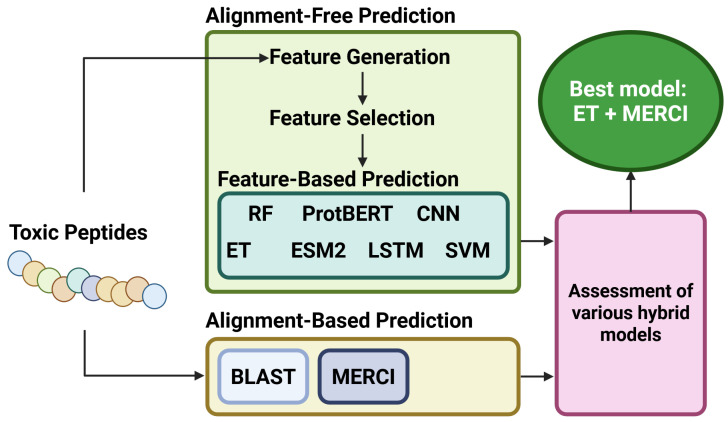
A schematic representation of the model optimization process for ToxinPred 3.0. A dataset of toxic peptides is used to optimize a variety of predictors and hybrid ensemble prediction models for the ability to predict peptide toxicity. The various ensemble methods were assessed to find the most effective ensemble model, which was determined to be the ExtraTrees model coupled with the MERCI motif-based sequence analysis technique. Figure produced in Biorender.

When discussing peptide toxicity prediction, the inclusion of ToxinPred is necessary. The most recent installation, ToxinPred 3.0, is a hybrid model combining Extra Tree (ET) and Motif-based prediction models. The authors, Rathore et al., also rigorously test a variety of other configurations of hybrid ML structures, each with its own benefits and limitations. ET models are very similar to RandomForest (RF) models, but have the advantage of being slightly faster and less computationally demanding, as well as having some small distinctions in their decision-making process. Largely, both models are ensembles of multiple decision trees, which allow the model to sort new data into categories based on the optimized data splitting thresholds. For this article, it is enough to regard both ET and RF as ensembles of decision trees. Rathore et al. determined that there are several highly successful hybrid model configurations, but the most successful hybrid configuration was the combination of an ET binding predictor and the pattern model Motif—EmeRging and with Classes—Identifier (MERCI), originally invented by Vens et al. in 2011 [59]. The MERCI model uses a Perl script to search for motifs in a set of sequences using default parameters. Rathore et al. were able to identify 117 motifs that were unique to toxic proteins, which they then used to assign additional weights to the model predictions. By assigning motif-based weighting to the predictive ensemble, the model’s performance is improved. A schematic representation of how the ET + MERCI model was determined to be the optimized model configuration is illustrated in Figure 12.

As shown from the schematic in Figure 12, Rathore et al. determined that the best model of all the hybrid models tested was ET + MERCI. This was the finalized implementation of ToxinPred 3.0, which was able to achieve a sensitivity of 0.92, a specificity of 0.93, and an accuracy of 0.93 over the tailored ToxinPred 3.0 validation dataset. The ToxinPred 3.0 dataset is larger than the benchmark sets used by previous models, containing 5518 unique toxic proteins/peptides. Rathore et al. also tested ToxIBTL over their larger dataset, and found that the ToxIBTL sensitivity, specificity, and accuracy over the larger set were 0.70, 0.89, and 0.87, respectively. This is lower than the reported values from the original ToxIBTL manuscript. In particular, the sensitivity of ToxIBTL seems to suffer, with a reported −0.265 decrease over the larger set. This may imply that the ToxIBTL, while suited to the dataset it was trained on, may not be as effective in preventing false positives as ToxinPred 3.0. Given the increased size and diversity of the ToxinPred 3.0 dataset, and the improved metrics of ToxinPred 3.0 over the larger set, future researchers should select data based on the methods suggested by Rathore et al.

#### 5.2.5. PLPTP

The Protein Language Model for Peptide Toxicity Prediction (PLPTP) [60] is a hybridized ML model which incorporates Evolutionary Scale Modeling (ESM2), double-layer Bi-LSTM, and DNN structures to produce the final peptide toxicity prediction. This model was proposed to have a particularly high degree of success in predicting toxicity from imbalanced training sets, as compared to older models. The PLPTP model was trained and validated over a set of 2138 toxic peptides and 5375 non-toxic peptides. The set was split into 85%/15% for training and validation, respectively.

**Figure 13:**
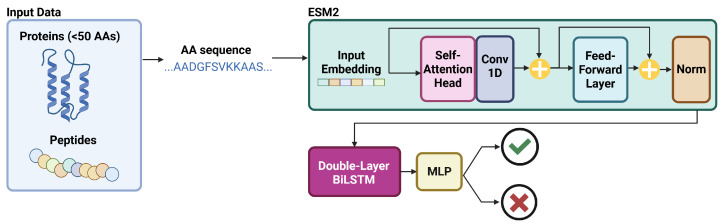
A schematic representation of the model architecture for PLPTP. Longer proteins and short peptides are fed into an ESM2 module, which embeds the inputs, and then runs the inputs through a self-attention head, a 1D convolution, and a feed-forward operation before being normalized and fed into a Double-Layer BiLSTM. The resultant output from the BiLSTM is then fed to an MLP module, which makes the final prediction as to whether a protein or peptide sequence is toxic. Figure produced in Biorender.

The PLPTP model begins by using the ESM2 model to extract relevant features from the input sequences, of which short-range and long-range amino acid dependencies can be observed. The ESM2 is also pre-trained on large-scale protein sequence data, to allow the model to encode evolutionary and structural insights that may influence peptide toxicity. The ESM2 model produces a sequence feature output vector, which is then fed directly into the double-layer Bi-LSTM, which captures forward- and backward-dependencies within the sequence. Following the Bi-LSTM module, the sequence feature vector is fed into a DNN, which transforms the data into the probability vector of peptide toxicity. The representation of this ML ensemble structure is shown in Figure 13.

The integration of these three modules serves to make the model capable of developing complex peptide sequence characteristics, and yields highly successful model predictions. The use of ESM2 allows for the peptide features to be rigorously canvassed, the double-Bi-LSTM allows for ordering dependencies to be accounted for, and the use of DNN to establish the final prediction allows for complex data relationships to be observed. The final PLPTP model was able to achieve a sensitivity of 0.975, a specificity of 0.978, and an accuracy of 0.997, all of which are marked improvements over previous models.

#### 5.2.6. HyPepTox-Fuse

**Figure 14:**
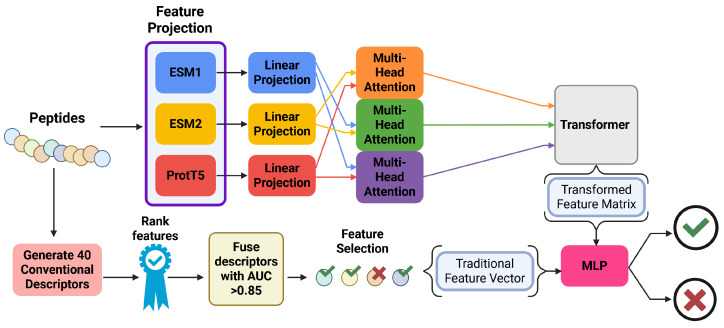
A schematic representation of the model architecture for HyPepTox-Fuse. Like other conventional models, 40 conventional descriptors are extracted directly from the sequence, ranked, optimized, and fused into a traditional feature vector. In parallel, sequence data is fed to several projection modules, which engage in crosstalk to determine the effect of module differences in predicting peptide toxicity. The outputs from the multi-head attention modules are then fused into a transformed feature matrix via a transformer. The traditional feature vector and the transformed feature vector are then concatenated into a single fused feature vector, and fed into an MLP module to make the final peptide toxicity prediction. Figure produced in Biorender.

Many toxicity prediction models focused on the integration of sequence-based features and some structural characteristics. Tran et al. take this concept of sequence-based learning a step further by presenting HyPepTox-Fuse, which meshes a protein language model (PLM) with conventional peptide descriptors to enhance the quality and amount of data available to the model. Similar to PLPTP, HyPepTox-Fuse also utilizes ESM2, along with ESM1 and ProtT5 to produce language-based feature projections of the dataset peptides. These language-based feature vectors are then assessed together via multi-head attention (using the keys and values from another method to answer the queries from a specific model), and fused into a single language-based classifier input vector via a transformer module. In parallel to this language processing step, conventional descriptors such as AAC, DPC, and other features are extracted from the peptide sequences as has been done in other studies. These conventional features are then ranked by the degree of impact on peptide toxicity prediction, and the most influential features are then vectorized to be fed into the classifier. Once the language processing vectors and the conventional descriptor vectors are prepared, they are concatenated into a single input vector and fed to the classifier module, which produces the final predictions of toxic or non-toxic. A schematic representation of this process is represented in Figure 14.

HyPepTox-Fuse demonstrated comparable performance with other models with a sensitivity, specificity, and accuracy of 0.883, 0.930, and 0.905 on the ToxinPred 3.0 dataset, respectively. Additionally, due to the inclusion of three distinct language-based predictors rather than only ESM2, the HyPepTox-Fuse model demonstrated a greater robustness and generalizability compared to previous models. Over the ToxTeller dataset, the performance of HyPepTox-Fuse only decreased in accuracy by four percentage points, compared to the eight point drop observed in ToxIBTL. This indicates that the inclusion of multiple language predictors slightly improves the robustness and generalizability of the HyPepTox-Fuse model over prior models.

#### 5.2.7. Comparison of Models

In terms of dataset biases, the cited toxicity models are well-suited to minimize biases—almost all have equal compositions of toxic and nontoxic datapoints, and they have a reasonable distribution of peptide lengths and protein lengths, where applicable. ATSE produced a novel dataset by querying public databases, yielding 1932 toxic and 1932 nontoxic peptides. The successor model, ToxIBTL, uses the ATSE dataset in tandem with the protein toxicity dataset ToxDL [61], which allows the model to be trained primarily on the protein toxicity dataset (that has more datapoints), and then fine-tuned on the smaller ATSE peptide toxicity dataset. This allows for any potential biases due to the smaller size of the ATSE set to be mitigated slightly. The tAMPer model also uses ToxDL in tandem with an in-house hemolysis dataset to train a toxicity predictor – this could bias the model towards accurate prediction of peptides that cause hemolysis, rather than other kinds of toxicity. However, it is observed that the tAMPer model still performs well against a benchmark dataset, so this is likely not a significant source of model bias.

ToxinPred 3.0 proposed a new, larger dataset, including 5518 toxic and 5518 non-toxic peptides. The increased size of ToxinPred 3.0 is useful, as it allows for any potential biases due to the small size of the ATSE set to be minimized by the addition of more training data. HyPepToxFuse also uses this dataset. PLPTP proposed another novel dataset, combining CSM-Toxin, ToxinPred 2.0, and ATSE datasets to form a massive set, and then eliminating several hundred datapoints by restricting the dataset to peptides less than 50 amino acids in length, composed only of the twenty natural amino acids. Because of the large volume of the initial set, PLPTP can afford to narrow the scope of the data being used for training, which will make the model more adept at performing that narrower predictive task (predicting toxicity of short- to medium-length peptides composed of natural amino acids). However, the curated PLPTP set is not balanced: there are 2138 positive peptides and 5375 nontoxic peptides. This could bias the dataset towards predicting that a given candidate peptide is nontoxic, which would increase the rate of false negatives. This is a problem if not sufficiently addressed, because if peptides are falsely predicted to be non-toxic and then progressed to further screening, they will fail at the in vitro or in vivo stages rather than the in silico stage, wasting time and money. However, the authors of PLPTP assert that they have performed rigorous statistical analysis of the amino acid frequency distribution and peptide length distribution over the dataset, and they have concluded that the frequency of certain amino acids is distinct between toxic and non-toxic peptides, and that the distribution of peptide lengths is uniform across the training and testing sets. Therefore, they propose that the presence of critical amino acids in the toxic and non-toxic peptides play a key role in PLPTP identifying peptide toxicity, and that the dataset imbalance is not a significant source of model bias.

Each of these models have a suitable amount of bias minimization: all excepting PLPTP have well-balanced datasets in terms of toxic and non-toxic datapoints, and PLPTP has demonstrated that their imbalanced set is not leading to undue bias towards non-toxic predictions. Further, the length of peptides is relatively uniform across training and testing sets for each of the cited models. In terms of the risk of labeling noise, most models are restricted to experimentally-validated toxicity data. Therefore, the likelihood of label noise negatively affecting the model performance of any of the cited models is low. However, it should be noted that tAMPer also relies on the predicted 3D structure of AMP datapoints as predicted by ColabFold. Since this cannot be a perfect prediction, this introduces the possibility of label noise, as the ColabFold model, if incorrect, could detrimentally effect the model’s ability to correctly predict the toxicity of a given AVP. The tAMPer model, while it does use Colab to predict structural data, does not have a very high risk of data leakage, as ColabFold is a physics-based model where tAMPer is a features-based model. Therefore, it is unlikely that the attributes and datapoints used to train each model overlap in a meaningful way, so the risk of data leakage affecting tAMPer’s performance is minimal. The other models do not have a high risk of data leakage: each model definitively separates the training and testing data, to ensure that there is no overlap, so any data leakage concern is kept to a minimum.

When selecting a peptide toxicity model to screen candidate peptide sequences, PLPTP should be selected if the candidates fall within the dataset constraints for that model (linear, less than 50 natural amino acids). This is because PLPTP had the best model performance of the cited models. However, if a more generic technique is needed, ToxinPred 3.0 or ToxIBTL are likely to be the most suited—although ToxIBTL technically has better performance metrics than ToxinPred 3.0, it also has a much smaller dataset. This indicates that its improved performance could be due to overfitting, which would make ToxinPred 3.0 the more robust choice of the two.

### 5.3. Clinical Adverse Event Prediction

When designing a novel AVP, arguably the most crucial step is to minimize as much as possible the adverse drug events (ADEs) associated with the novel drug—the positive benefits of the treatment must not be overshadowed by harm caused to the patient. To date, postmarketing surveillance of drug ADE reporting has been used to identify ADEs in a more diverse patient population than is possible in a clinical study. This is usually accomplished with signal detection algorithms—low-level computer programs that are capable of counting up the amount of ADEs present for a specific drug, and comparing the result to the number and type of expected ADE reports for a ‘safe’ drug. If the number of reported ADEs is significantly higher than expected, then the signal detection algorithm will flag a drug as being potentially problematic, pending more rigorous study of the ADE that is of concern [62]. While this method of scanning postmarket data does identify ADEs after the drug has gone to consumers, it would be much more preferable to be able to accurately predict and flag potential ADEs with a high degree of specificity, sensitivity, and accuracy before the drug goes to market. To accomplish this, predictive ML is necessary. However, there are several barriers to the effective use of ML in ADE prediction. Primarily, the twin issues of sampling bias and reporting bias are extremely prevalent in ADE data. This imprecision of the available ADE data has led to much of the research efforts in minimizing drug ADEs towards predicting and minimizing drug toxicity, rather than directly predicting ADE presence or absence. Unfortunately, a lack of a predicted toxicity response is not always indicative of a lack of ADEs associated with a novel drug [49]. Therefore, the increasing power and reliability of drug toxicity prediction, while helpful, does not fully solve the problem of minimizing ADEs. To do this, it is necessary to develop a robust database of ADE datapoints with the impacts of confounding variables smoothed out.

To accomplish this, several methods have been proposed to somewhat mitigate the gaps in ADE reporting. Information such as patient demographic and treatment application conditions are not uniformly reported, resulting in the individual researcher interpreting clinical study data, which will impact the resultant predictive model [62,63]. The most recent database of ADE information, CT-ADE [63], encompasses 2497 drugs and 168,984 drug-ADE pairs from clinical trial results and accounts for confounding variables such as treatment and target population data, including conditional data such as dosage and administration route. The inventors of CT-ADE were also able to demonstrate the promising ability of various generalized classifier models to predict ADEs in a monopharmaceutical environment with a sensitivity and specificity higher than would be random chance, indicating that it would be possible to develop a classifier model that could predict ADE of a specific AVP before market release. This would be a great improvement over the current approach, and would significantly reduce the capacity for patient harm.

As the CT-ADE predictive model relies on reported patient data, there is a much greater opportunity for labeling noise—patients may under-report symptoms, or providers may document symptoms improperly, leading to the mislabeling of data. This would lead to label noise, and could result in ADE-triggering AVPs not being labeled as ADE-causing. This would by extension harm the model’s predictive capabilities. However, CT-ADE minimizes the risk of improper documentation by strict gating of what ADE data was included in the dataset, limiting datapoints to only include extremely well-documented ADEs. Further, confidence intervals were assigned to each data point. Each datapoint was assigned a value of 1 (ADE-causing) if the authors were 95% certain that at least 1% of the patient population would experience that ADE, and 0 otherwise. This strict screening and data-gating helps to minimize the risk of labeling noise for the CT-ADE dataset, and improves the overall confidence in the classifier models proposed by the CT-ADE authors. Within the CT-ADE dataset, the most commonly occurring ADEs are gastrointestinal disorders (38.33%), nervous system disorders, and infections, so the model may be slightly biased towards correctly predicting those ADE categories, but the overall performance of classifiers proposed by the authors of CT-ADE does not appear to suffer due to this.

**Figure 15:**
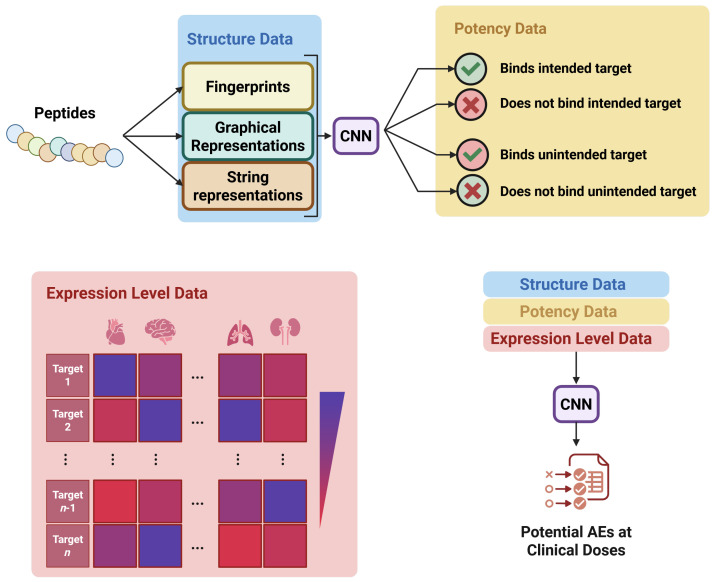
A schematic representation of the model architecture for the ADE predictive model proposed by Cao et al. From the peptide sequence, structure-based features are extracted and fed through a CNN and used to predict potency data about each peptide. In tandem with this, expression level data about the level of target protein expression in critical organs is tabulated and condensed into an input vector. The structure, potency, and expression data are then fed in combination to a CNN module, which makes a predicted list of the likelihood of various AEs at a clinical dose level. Figure produced in Biorender.

In addition to predicting ADEs from ADE reporting databases such as CT-ADE, there is also some interest in predicting ADEs more indirectly. Cao et al. [49] developed a two-part model which first determines the IC50 value between a drug and a wide variety of potential targets, and then feeds the resultant predicted IC50 value vector to a secondary model which predicts ADE presence and type based on the IC50 predictions. This is an interesting approach—IC50 value can be predicted with a high degree of sensitivity, specificity, and accuracy, as seen in the various toxicity prediction models, however the integration of a secondary predictive model to tie the IC50 values to a predicted ADE is highly successful, with the ADE predictive model able to predict the presence or absence of headaches, nausea, and diarrhea with a sensitivity, specificity, and accuracy all above 90%. The schematic representation of the tandem ML model used by Cao et al. is depicted in Figure 15.

The Cao et al. model uses a subset of the SIDER [64] database, which includes 759 drugs and 994 side effects. It is a slightly older dataset, as it was published in 2016 whereas CT-ADE was published in March 2025. SIDER utilizes natural language processing (NLP) to scan package inserts and other documentation for reported possible ADEs. The use of ML to locate and define positive ADE datapoints introduces a high level of risk for label noise—the NLP will make a little mistake inevitably, and this will deteriorate the quality of the obtained ADE data. The authors of SIDER admit freely that there is a high risk of false negatives being present, with 28,343 of the 140,064 (20%) of the drug-ADE pairs in SIDER 4 able to be successfully matched to known drugs and diseases in a publicly available dataset. However, the probability of a compound being falsely labeled positive (an indication being misreported as an ADE) is extremely low at 1.2%. These results indicate that while there is a high rate of false negatives in the NLP-based data sourcing method used by SIDER, the large subset of datapoints which can be traced to known drugs and diseases is most likely credible. Further adding to the credibility of the Cao et al. model is the fact that the model dataset is constrained to drugs with known monotherapeutic ATC codes, which may improve the label noise over the original SIDER dataset, although it drastically decreases the dataset size.

In light of these issues, the Cao et al. model likely has a reasonable capability for predicting the most common clinical ADEs for a given compound. However, the strong risk of label noise coupled with the lack of data for more obscure ADEs leads to a slight lowering confidence in the model’s outputs.

Overall, the prediction of ADE presence, type, and strength from databases like CT-ADE remain a challenging but fascinating prospect. The model by Cao et al. represents an effective predictive model over the curated subset of SIDER utilized for training and validation. That dataset was small in comparison to larger ADE sets like CT-ADE—there were only 759 drug compounds and 477 protein targets, compared to the 2497 drugs and 168,984 drug-ADE pairs within ADE. This indicates that while the results from Cao et al. are promising, it will be critical to base future ADE classifiers on the CT-ADE dataset, as they have a larger volume of strictly controlled, up-to-date information.

### 5.4. Generation of Novel AVP Sequences

In addition to the classification of AVP candidates as binding or non-binding, and toxic or non-toxic, there is also an emergent interest in synthetically generating the initial candidate AVP amino acid sequence, even before the prediction of binding or toxicity behaviors. Through the implementation of Generative Pretrained Transformers coupled with a large database of AVP sequence information, a model can be constructed that is capable of predicting a successful AVP sequence purely in silico, rather than through traditional lead identification steps. There are several obvious benefits to this—by implementing in silico lead identification in the form of a GPT model, a great deal of time and material costs can be minimized, as well as the potential for maximizing the value of already existing data by using prior experimentation to enhance the GPT’s predictive ability. However, there are some pitfalls which need to be carefully guarded against when implementing GPT. Primarily, it is critical to be cautious of bias within the pretraining dataset. If there are duplicate instances within the pretraining dataset, such as the same AVP sequence appearing more than once, or a large study with several near-identical sequences is used to supplement the pretraining set, this will make the GPT much more likely to propose that shared sequence fragment as part of the new proposed AVP sequence. To mitigate this, it is critical to carefully select the GPT pretraining set to be as fair a representation as possible of the AVP chemical space, and to ensure as high a degree of diversity within the pretraining set as possible, to allow the GPT to form a diverse vocabulary on which to base its predictive generation.

This method of GPT prediction is largely novel to the field of AVP development. The recent publication of Antiviral Peptide-Generative Pre-Trained Transformer (AVP-GPT) by Zhao and Song [65] is, to the best knowledge of the authors, the first generative AVP sequence model in the literature. With this in mind, we will examine the achievements and future goals outlined by Zhao and Song, to assess the current status and future prospects of AVP sequence generation via GPT.

Broadly speaking, highly successful GPT models rely on two training components: pretraining and fine-tuning. Pretraining a GPT model involves vectorizing a large body of relevant data and submitting it to the GPT model, to inform the weights involved at the different model layers within the larger model. This allows the GPT to form similar output vectors to the inputs that are found in the pretraining body of data. In the case of highly specialized GPTs, such as AVP-GPT [65], a fine-tuning step is also critical. This is when, after pretraining has taken place, the GPT is fed a smaller volume of more relevant datapoints, to finalize the weights of the model to perform best on the fine-tuning dataset, using the patterns learned from the pretraining dataset to fill any gaps which result from the fine-tuning dataset potentially being limited in size. This process of pre-training and then fine-tuning data allows for a highly effective GPT model to be trained on a limited amount of data, provided a larger volume of similar-looking data is also available.

**Figure 16:**
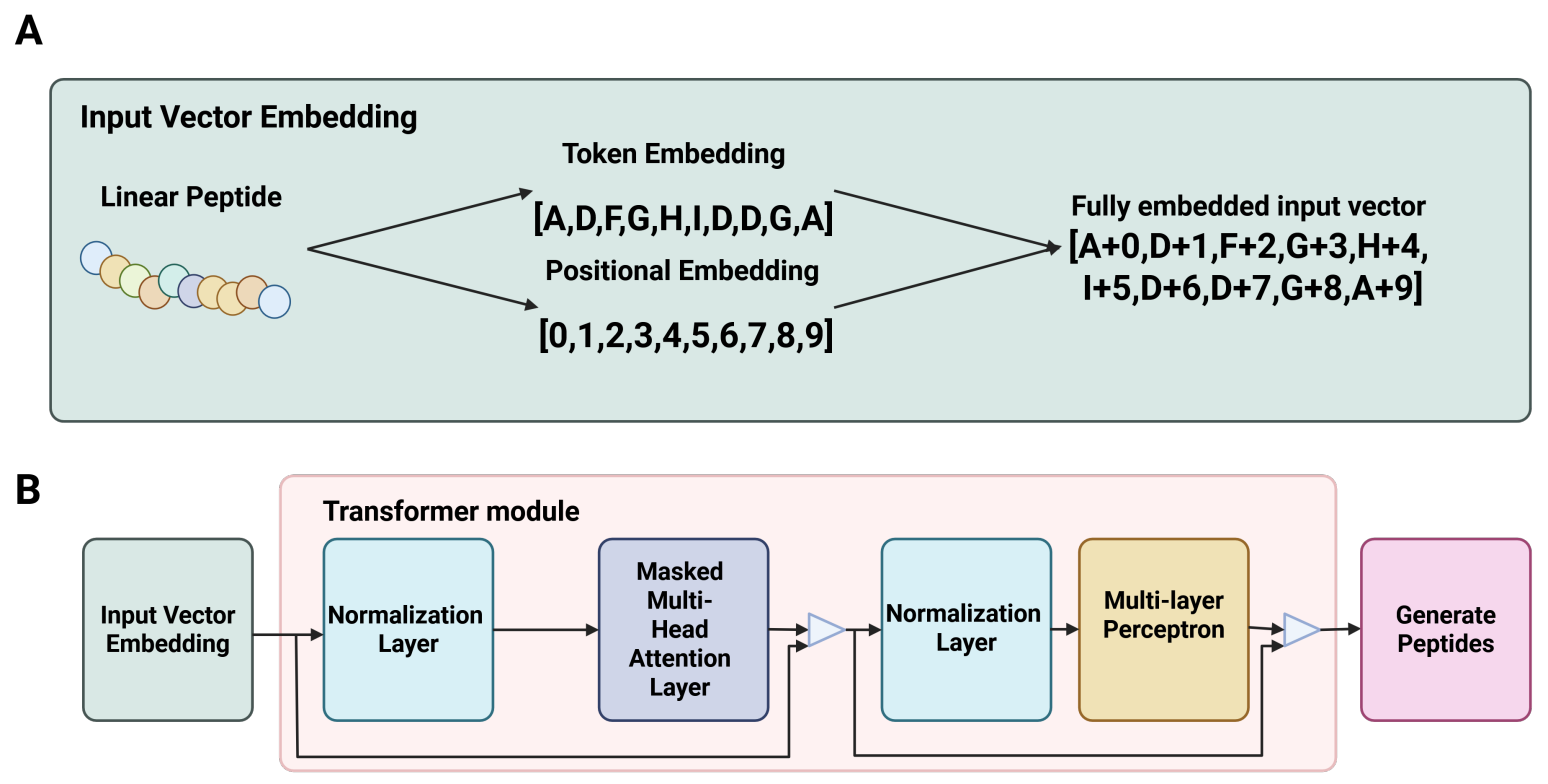
(**A**) A schematic representation of how a linear peptide is encoded into an input vector for AVP-GPT. (**B**) A schematic representation of the generator portion of AVP-GPT’s architecture for the AVP-GPT model. Figure produced in Biorender.

AVP-GPT is the first published application of a generative model to predict novel AVP sequences. For AVP-GPT, Zhao and Song used the larger volume of RSV-targeting AVP sequences which were available to them to pretrain AVP-GPT, and then used a smaller volume of AVP sequences targeting INFVA, HPIV, SARS-CoV and SARS-CoV-2 to fine-tune the model. As illustrated in the schematic, datasets from AVPdb and DRAVP were combined with proprietary Youcare data available to Zhao and Song to form a larger dataset of AVP sequences tagged with 0 for non-AVP behavior and 1 for AVP behavior. The sequences were also sorted by the disease target, to allow for the separation of pretraining and fine-tuning data. Following this tagging process, the AVP sequences were tokenized into smaller sequence fragments, as well as analyzed for descriptors and fingerprints, and the relevant disease receptors were tokenized into smaller sequence fragments. Following this step, the data is vectorized via a combination of transformer encoders and single-layer CNNs to create one-dimensional vector datapoints, each representing the full breakdown of a peptide-receptor pair. At this point, vectors which held RSV-targeting AVP data was used to pre-train the GPT by sequential generation, classification, and self-correction steps. Classification is possible by withholding a small subset of the positive data from each disease target, along with the full body of negative (non-AVP) data from each disease category. This subset can then be used to classify the generated peptide sequences in silico, which is how the GPT model can perform self-correction and improve by adjusting the weights while in the pretraining and fine-tuning stages. Representations of the peptide input embedding process, as well as a schematic of the generator portion of AVP-GPT’s architecture, are shown in Figure 16.

After the full body of RSV-targeting AVP sequences had been utilized, the same process was carried out in the fine-tuning step with the INFVA/HPIV/SARS-CoV/SARS-CoV-2 data, where the model generated, classified the generated output, and self-corrected based on the difference between predicted and actual output.

The pretrained AVP-GPT model demonstrates an ability to propose AVP sequences of a high quality, with a sensitivity, specificity and accuracy of 0.875, 0.940, and 0.915 when classifying the generated peptides in silico to assess their AVP potential against RSV. The pretrained model, before fine-tuning, was also able to generate a low perplexity. In machine learning, perplexity is a metric which is used to determine the reliability of generated data, and is calculated by the exponent of the loss of the model. This metric is common when discussing GPT models, as it is a good frame of reference for how reliable the model output is, alongside the sensitivity, specificity, and accuracy of the classifier portion of the GPT. Lower perplexity indicates a higher confidence in the quality of the results, while high perplexity indicates the opposite. In this case, AVP-GPT was able to achieve a perplexity of 2.09 in the pretrained state. This is very low, compared to a similar method using LSTM, which was able to achieve a perplexity of 16.13. Overall, the model demonstrates a strong ability to produce novel AVP sequences with a high likelihood of interacting with the RSV target protein.

After the fine-tuning phase, the model was assessed again for its ability to generate strong disease-targeting AVPs, with special attention being paid to its ability to produce AVPs that target INFVA, HPIV, SARS-CoV or SARS-CoV-2. However, while there was comparable sensitivity, specificity, and accuracy of the AVP-GPT model post-fine-tuning compared to the pretrained model configuration, the model also experienced a large jump in perplexity, with model perplexity ranging from 7.41 to 10.18 depending on the disease target being examined. This indicates that there may be a lower confidence in the quality of the AVP sequences generated by the model to target non-RSV virus proteins, as compared to those designed to target RSV.

In addition to being able to achieve a strong predicted antiviral activity from the generated peptides, AVP-GPT can also generate a large volume of data at a rapid pace—it was able to generate 10,000 novel peptides over 2 days. Of those 10,000, 25 of those generated peptides were predicted to have a probability of RSV AVP activity above 90%. This subset of 25 was tested in vitro, and it was determined that 19 out of 25 were able to demonstrate an EC50 value less than 10µM with the associated RSV target protein. This is a promising outcome at a rapid rate of turnaround, illustrating the power of GPT in early-stage drug development when applied properly.

## 6. Current Challenges of AVP Design

In tandem with the mounting revolution of utilizing ML in the early stages of AVP design, there are still some practical limitations to AVP utility in a clinical application. By using in vitro and in vivo experimental data to validate the performance of ML models, the predicted results can be assigned a certain degree of confidence depending on the model’s accuracy, specificity and sensitivity. If the model predicts the real-world validation data well enough, the predicted best candidates can be confidently progressed to in vitro and in vivo testing stages. However, while the use of in silico predictive tools improves the odds of a selected candidate performing well on in vitro and in vivo testing, it does not guarantee it: the biological targets being examined may have a degree of complexity that is difficult to fully capture, which would reduce the efficacy of ML. By using in silico tools to thin the candidate pool prior to in vitro and in vivo testing, the failure rate at later stages may be reduced, as only predicted high-performing peptides are progressed to wet-lab testing. This is seen in the case study for AVP-GPT, where 19 out of 25 predicted candidates performed well in the in vitro stage. The primary utility of ML in early-stage drug design is to ensure that the risk of an AVP failing due to a lack of activity, high toxicity, or ADE presence is minimized prior to investing wet-lab resources, but the treatment regimen and mitigating external variables may still stymie AVP development at the early clinical stages even if the AVP was predicted to be a favorable candidate. To mitigate potential failure at that stage, there is an ongoing body of research dedicated to enhancing AVP effectiveness through structural modifications or combinations with other compounds to improve stability, encapsulation to protect the AVP from degradation in vivo, and other such practical innovations.

In addition to bridging the gap from preclinical testing to the early stages of clinical application, the acquisition of a sufficient volume of high-quality data is still difficult for AVP-specific binding, toxicity and ADE prediction. To further the goals of the larger scientific community, it would be highly beneficial if relevant AVP data repositories were de-privatized to make the best use of them in the broader body of research. Additionally, to facilitate the easy reference and comparison of models we have provided an itemized list of the various models and their respective tasks, which can be seen in Table 2.

## 7. Conclusions

ML is a valuable tool for pre-screening antiviral peptide candidates for valuable qualities, such as high target binding affinity and low cytotoxicity and cross-target binding, however there are still limitations in predicting ADE presence and degree of severity. Future directions for research in this field should include the de-privatization of privately-owned experimental AVP data, and the development of a diverse, updated benchmark dataset. It may also be highly useful to separate this dataset by target protein and virus type, to allow for the qualities of different AVPs to be distinguished. Further, a standardized development pipeline should be established, where candidates progress from in silico to in vitro and in vivo testing in a way that results in a robust body of data in support of the candidate AVP’s efficacy prior to clinical assessment. By mindfully implementing in silico tools in the early stages of AVP development, initial design and screening of AVPs can be executed at a higher rate and lower cost than traditional methods have allowed for. Furthermore, the use of ML in designing antiviral peptides opens up the field of antiviral drug design to researchers at lower BSL ratings, expanding the knowledge base of the field as a whole and accelerating the development of novel treatments for dangerous diseases.

## 8. Machine Learning Terminology

When it comes to machine learning, there are several useful descriptors for models that may not be obvious to the beginning ML user. For this reason, we will briefly lay out a series of vocabulary terms in Table 3 for ease of reference and understanding in the rest of the paper.

## 9. Database References

There are a large number of databases referenced in this review, each of which has its own merits and disadvantages for use in ML prediction of binding affinity, toxicity, and ADE presence and severity. Three tables are listed here to provide the reader with a complete repository of the available AVP databases, to avoid detracting from the momentum of the main body of text. Table 4 is a list of binding affinity and/or viral activity databases for AVPs, Table 5 is a list of toxicity data for general peptides, and Table 6 is a list of ADE databases which are publicly available.

## Figures and Tables

**Table 1:** Table of commonly-used sequence-derived sequence variables.

Variable	Abbreviation	Definition
Single Amino Acid Composition	AAC	Makeup of the peptide in terms of amino acids.
Dipeptide Composition	DPC	Makeup of the peptide in terms of pairs of amino acids.
Pseudoamino acid Composition	PseAAC	A representation of AAC that takes positioning of each amino acid into account.
K-spaced Amino Acid Pairs	KSAAP	Composition of the peptide as determined by pairs of amino acids separated by k many amino acids between the two elements of the pair.
Encoding Based on Group Weight	EBGW	Categorical sequence-derived features which are changed to a representation suitable for ML (numerical).
N5C5 Analysis	N5C5	AAC of the five amino acids around the N- and C-terminals of the peptide sequence.

**Table 2:** Summary of machine learning (ML) models discussed in this review for antiviral peptide (AVP) discovery and preclinical screening.

Model	Primary Task	Input Type	Dataset	Validation	Limitations
iAVPs-ResBi [9]	Binary binding affinity prediction	Sequence-derived peptide features	AVPdb [66]	4-fold testing: 95% accuracy on all four subsets	Performance saturates with network depth; relies on existing curated data (limited diversity).
VEIP Predictor [28]	Predict binary VEIP activity	Sequence-derived features from peptides and target viral envelopes	246 VEIPs vs. 246 non-VEIPs from DBAASP [55], UniProt [67]	73/27% train/test split; RealAdaBoost yielded 0.90 accuracy on test set	Small dataset limits generalizability.
FIRM-AVP [54]	Binary binding affinity prediction	Sequence-derived features, reduced by feature selection	AVPpred [56]	10-fold cross-validation (SVM achieved 92.4% accuracy)	Small, outdated dataset.
ATSE	Binary toxicity prediction	Molecular graph of predicted 3D peptide structure and evolutionary profile (PSSM), both sequence-derived	Collected peptide toxicity dataset [57]	5-fold CV on training data; independent test (reported Sn/Sp/Acc 96/94/95%)	No transfer learning used, may struggle with generalizability (addressed by successor model ToxIBTL).
ToxIBTL [58]	Binary peptide toxicity prediction (with transfer learning)	Same multimodal inputs as ATSE [57], but employs pretrained model on large protein toxicity data, then fine-tunes on peptide data	Large protein toxicity dataset for pre-training (size: many thousands of proteins) + peptide toxicity dataset for fine-tuning (e.g., 2–3 K peptides) [58]	Trained in two stages; evaluated on peptide test set – improved Acc 0.96, Sn 0.963, Sp 0.954 vs predecessor	Requires extensive protein data in pre-training, increased complexity over predecessor model.
tAMPer [68]	Binary peptide toxicity prediction and secondary structure prediction	Multi-modal: amino acid sequence + predicted 3D structure graph (ColabFold-derived) fused via Bi-GRU and GNN; outputs both toxicity classification and secondary structure traits	Same peptide toxicity set as ToxIBTL [58] plus additional in-house hemolysis data	5-fold CV; external data integration (showed +0.03 F1 improvement over ToxIBTL)	Slight F1 boost, but did not report direct Acc/Sn/Sp for comparison. 3-D prediction adds computational cost.
ToxinPred 3.0 [69]	Binary peptide toxicity prediction	Sequence-based features and motif patterns: ExtraTrees ensemble classifier combined with MERCI motif-identification weighting	ToxinPred3 dataset—5518 unique toxic peptides/proteins (augmented larger benchmark)	External validation on held-out set; achieved 92% Sn, 93% Sp, 93% Acc on its curated dataset	Model performance tied to identifiable motifs; may miss toxic peptides lacking known motif patterns.
PLPTP [60]	Binary peptide toxicity prediction	Hybrid: Transformer-based protein language embeddings (ESM-2, etc.) + traditional descriptors, fused into MLP classifier	7513 peptides (2138 toxic + 5375 non-toxic) compiled from multiple sources	Model attained 97.5% Sn, 97.8% Sp, 99.7% Acc on 5-fold CV, plus comparison on independent test sets	Extremely high apparent accuracy may indicate overfitting. Motif analysis improves interpretability, but requires large transformers that raise computational requirements.
HyPepToxFuse [70]	Binary peptide toxicity prediction	Multi-head fusion of sequence embeddings and features: uses multiple protein language models (ESM-1b, ESM-2, ProtT5) alongside 40 classic sequence descriptors, combined via attention and transformer layers	ToxinPred3.0 dataset for training (5.5 k peptides); tested on ToxTeller external set	5-fold CV on training data; independent test on ToxTeller dataset to assess generalization. ToxTeller test resulted in 88.3% Sn, 93.0% Sp, 90.5% Acc; only 4% accuracy drop on external test vs. 8% drop in prior model (ToxIBTL)	More complex architecture increases runtime but yields better robustness. Constrained by available toxicity labels—novel data or features required to significantly improve performance.
Two-Step ADE Model [49]	ADE prediction for peptide drugs	Multi-modal: peptide’s predicted multi-target potencies + tissue-specific target expression levels	Trained on CT-ADE database (168,984 drug—ADE pairs from 2497 drugs)	Evaluated on held-out clinical trial data; achieved >0.90 Sn/Sp/Acc for multiple adverse events, validated in a monotherapy setting	Clinical ADE data suffer from under-reporting, confounders. Model accuracy depends on comprehensive target profiling—may miss idiosyncratic or immune-mediated ADEs. Requires large, curated datasets (CT-ADE); still limited for peptide therapeutics.
AVP-GPT [65]	Generative AVP design (de novo antiviral peptide generation)	Transformer-based generative model: a GPT fine-tuned on known AVPs, conditioned on virus target context. Model generates candidate peptide sequences, then iteratively self-filters them via an internal classifier for antiviral probability	10 k known peptide sequences labeled for antiviral activity against various viruses (combined public AVP databases + proprietary YouCare data)	Internal validation via perplexity (text-generation metric) and an integrated classifier. After training (pre-trained on RSV-target AVPs, fine-tuned on other viruses), the model produced 10,000 novel peptides; 25 top candidates were synthesized, with 19 showing sub-10 µM EC50 in vitro against RSV. Fine-tuned model achieved 91.5% accuracy in silico for RSV-targeted AVPs. Post-finetuning performance remained high for other viruses, though perplexity rose.	Lower confidence targeting under-represented viruses—perplexity jumped from 2 to 7–10 when generating for non-RSV targets, indicating potential generalization limits. Generation focuses on antiviral activity; stability, toxicity are not explicitly optimized, so additional filtering is needed before clinical consideration.

**Table 3:** Summary table of basic ML terminology.

Term	Definition	Example
Instance	All characteristic information about a single candidate peptide.	Sequence or structural information, target binding affinity, toxicity, hydrophobicity, charge, etc. A single data point in a dataset.
Dataset	A collection of many instances.	Publicly available datasets, such as in DRAVP.
Variable	A quality of the peptide that can be measured or observed.	Hydrophobicity, charge, structural orientation, peptide sequence, etc.
Target	The quality or qualities of the peptide being predicted.	Binding affinity, toxicity, etc.
Positive Instance	An instance where the binary positive outcome is observed.	If a dataset is reporting binding to a receptor, the positive instances would be every instance with a strong binding to the receptor.
Negative Instance	An instance where the binary positive outcome is not observed.	Negative instances would be every instance in the dataset not classified as a positive instance.
Accuracy	A ratio of correct classifications to the total number of classifications	A model may correctly predict 8 out of 10 positive instances and 7 out of 10 negative instances. This model has an accuracy of 15/20, or 0.75.
Sensitivity	A ratio between the number of predicted positive instances and the number of positive instances present.	A model may correctly predict 8 out of 10 positive instances, which would make the model sensitivity 0.80.
Specificity	A ratio between the number of predicted negative instances and the number of negative instances present.	A model may correctly predict 9 out of 10 negative instances, which would make the model specificity 0.90.
Perplexity	The calculated exponent of the loss observed when training a GPT: used to assess the level of confidence in the generated sequence output.	If the loss observed in the fully trained model is high, then the model’s performance is poor, and the perplexity of the model is very high.

**Table 4:** Summary table of databases with AVP binding affinity and/or viral activity data.

Database	Number of AVPs	Source
APD3	188	[71]
DRAMP 4.0	3681	[72]
AVPdb	2683	[66]
DBAASP	1535	[55]
CAMPR3	55	[73]

**Table 5:** Summary table of current publicly-available peptide toxicity data.

Database	Number of Peptides	Source
admetSAR 3.0	172,116	[74]

**Table 6:** Summary table of current publicly-available ADE data.

Database	Validated Drug-ADE Pairs	Source
CT-ADE	168,984	[63]
SIDER	28,343	[64]

## Data Availability

Data generated by the authors concerning the makeup of the AVPpred and VEIP datasets.

## Abbreviations

The following abbreviations are used in this manuscript:

AI	Artificial Intelligence
AVP	Antiviral peptide
ML	Machine Learning
AMP	Antimicrobial peptide
GWAS	Genome-wide association study
ADE	Adverse drug effect
RF	RandomForest
SVM	Support Vector Machine
NN	Neural Network
MLP	Multi-Layer Perceptron
DNN	Deep Neural Network
QSAR	Quantitative Structure-Activity Relationship
CNN	Convolutional Neural Network
GNN	Graph Neural Network
RNN	Recurrent Neural Network
GRU	Gated Recurrent Unit
BiGRU	Bidirectional Gated Recurrent Unit
LSTM	Long Short-Term Memory
BERT	Bidirectional Encoder Representations for Transformers
GPT	Generative Pre-trained Transformer
HTS	High-Throughput Screening
